# Evolution and Expression of Reproductive Transition Regulatory Genes *FT*/*TFL1* With Emphasis in Selected Neotropical Orchids

**DOI:** 10.3389/fpls.2020.00469

**Published:** 2020-04-21

**Authors:** Diego A. Ospina-Zapata, Yesenia Madrigal, Juan F. Alzate, Natalia Pabón-Mora

**Affiliations:** ^1^Facultad de Ciencias Exactas y Naturales, Instituto de Biología, Universidad de Antioquia, Medellín, Colombia; ^2^Centro Nacional de Secuenciación Genómica, Sede de Investigación Universitaria, Facultad de Medicina, Universidad de Antioquia, Medellín, Colombia

**Keywords:** flowering, *FLOWERING LOCUS T*, *TERMINAL FLOWER 1*, Orchidaceae, gene evolution

## Abstract

Flowering is a rigorously timed and morphologically complex shift in plant development. This change depends on endogenous as well as environmental factors. *FLOWERING LOCUS T* (*FT*) integrates several cues from different pathways acting as a flowering promoter. Contrary to the role of *FT*, its paralog *TERMINAL FLOWER 1* (*TFL1*) delays floral transition. Although *FT*/*TFL1* homologs have been studied in model eudicots and monocots, scarce studies are available in non-model monocots like the Orchidaceae. Orchids are very diverse and their floral complexity is translated into a unique aesthetic display, which appeals the ornamental plant market. Nonetheless, orchid trade faces huge limitations due to their long vegetative phase and intractable indoor flowering seasons. Little is known about the genetic basis that control reproductive transition in orchids and, consequently, manipulating their flowering time remains a challenge. In order to contribute to the understanding of the genetic bases that control flowering in orchids we present here the first broad-scale analysis of *FT*/*TFL1*-*like* genes in monocots with an expanded sampling in Orchidaceae. We also compare expression patterns in three selected species and propose hypotheses on the putative role of these genes in their reproductive transition. Our findings show that *FT-like* genes are by far more diversified than *TFL1-like* genes in monocots with six subclades in the former and only one in the latter. Within *MonFT1*, the comparative protein sequences of MonFT1A and MonFT1B suggest that they could have recruited functional roles in delaying flowering, a role typically assigned to TFL1-like proteins. On the other hand, MonFT2 proteins have retained their canonical motifs and roles in promoting flowering transition. This is also shown by their increased expression levels from the shoot apical meristem (SAM) and leaves to inflorescence meristems (IM) and floral buds (FBs). Finally, *TFL1-like* genes are retained as single copy and often times are lost. Their loss could be linked to the parallel recruitment of *MonFT1A* and *MonFT1B* homologs in delaying flowering and maintaining indeterminacy of the inflorescence meristem. These hypotheses lay the foundation for future functional validation in emerging model orchid species and comparative analyses in orchids with high horticultural potential in the market.

## Introduction

The transformation of a vegetative shoot apical meristem (SAM) into an inflorescence meristem (IM) forming bracts and floral buds (FBs) is a rigorously timed and morphologically complex shift in plant development (Henderson and Dean, 2004; Benlloch et al., 2007; Liu et al., 2009; Thouet et al., 2012). In the model species *Arabidopsis thaliana* (Brassicaceae) floral transition depends on endogenous as well as environmental factors, which have been dissected into different pathways. These include the photoperiod, vernalization, hormonal and autonomous age-related pathways (Mouradov et al., 2002; Blázquez et al., 2003; Mutasa-Göttgens and Hedden, 2009; Amasino, 2010). All routes converge in the floral integrators, a group of genes that control the vegetative-to-reproductive transition which include *FLOWERING LOCUS T* (*FT*), *SUPPRESSOR OF OVEREXPRESSION OF CONSTANS 1* (*SOC1*), *LEAFY* (*LFY*), *FLOWERING LOCUS D* (*FD*), and *AGAMOUS like 24* (*AGL24*) (Boss et al., 2004; Wellmer and Riechmann, 2010).

Within the photoperiod pathway, *FT* plays one of the most important functions in reproductive transition. *FT* is synthesized in the leaves and transported through the phloem, both as mRNA and protein, to the SAM, where it triggers flowering (Corbesier et al., 2007; Andrés and Coupland, 2012; Lu et al., 2012). *FT* overexpression results in early flowering plants with determinate inflorescences (Kardailsky et al., 1999). Conversely, *ft* mutant plants produce more leaves and have delayed flowering (Koornneef et al., 1991). In addition, *FT* is positively regulated by *CONSTANS* (*CO*), an upstream activator responsive to light exposure (Suárez-López et al., 2001; Valverde, 2011). In the SAM, FT interacts with FD and promotes the expression of *APETALA 1* (*AP1*) and *SOC1* (Abe et al., 2005; Wigge et al., 2005; Lee and Lee, 2010). In turn, *SOC1* and *AGL24* induce the transcription of *LFY* (Lee and Lee, 2010). Both *LFY* and *AP1* control floral meristem identity directly by repressing inflorescence meristem factors (Lee and Lee, 2010). Comparative studies in model monocots like *Oryza sativa* have found a third protein, 14-3-3 which mediates the interaction between OsFD1 (the *FD* homolog) and Hd3a (the *FT* homolog). The complex formed by these proteins is recognized as the florigen activation complex (FAC), a direct inducer of *OsMADS15* (the *AP1* homolog) (Taoka et al., 2011, 2013). Contrary to the role of *FT*, its paralog *TERMINAL FLOWER 1* (*TFL1*) delays floral transition (Ratcliffe et al., 1998). In *Arabidopsis*, overexpression of *TFL1* results in delayed flowering and indeterminate inflorescences (Ratcliffe et al., 1998, 1999). On the other hand, *tfl1* mutants exhibit short vegetative phases and develop inflorescences with terminal flowers (Shannon and Meeks-Wagner, 1991; Alvarez et al., 1992; Bradley et al., 1997; Ratcliffe et al., 1998). It has also been shown that *XAANTAL2* (*AGAMOUS-Like14*) positively regulates *TFL1* expression in the SAM to promote inflorescence identity (Pérez-Ruiz et al., 2015). Interestingly, TFL1 also interacts with FD, resulting in the negatively regulation of *AP1* and *LFY*. As a result, during the vegetative-to-reproductive transition a negative feedback loop is maintained between the inflorescence regulator *TFL1* and the floral meristem genes *AP1* and *LFY* (Ratcliffe et al., 1999; Conti and Bradley, 2007; Hanano and Goto, 2011).

Both *FT* and *TFL1* are members of the Phosphatidyl-Ethanolamine-Binding Protein (PEBP) family. In plants this gene lineage is divided into the *MFT-like*, *FT-like*, and the *TFL1-like* subfamilies (Hedman et al., 2009; Karlgren et al., 2011; Liu et al., 2016). In *A. thaliana*, several members of the PEBP family have been recognized as promoters [e.g., *TWIN SISTER OF FT* (*TSF*) and *MOTHER OF FT AND TFL1* (*MFT*)] or repressors [*ARABIDOPSIS THALIANA CENTRORADIALIS* (*ATC*) and *BROTHER OF FT AND TFL1* (*BFT*)] of the floral transition (Yoo et al., 2004, 2010; Yamaguchi et al., 2005; Huang N. C. et al., 2012). Comparative studies of *FT*/*TFL1* homologs have shown that their role in regulating reproductive transition is conserved in Cucurbitales (Lin et al., 2007), Fabales (Nan et al., 2014), Gentianales (Imamura et al., 2011), Poales (Tamaki et al., 2007), Rosales (Randoux et al., 2014), and Solanales (Hayama et al., 2007). Nevertheless, other roles have also been reported for *FT*/*TFL1* homologs including associated changes in leaf shape and size during flowering in *Solanum lycopersicum* (Lifschitz et al., 2014), tuber development in *Solanum tuberosum* (Navarro et al., 2011; Teo et al., 2017), and bulb formation in *Allium cepa* (Lee et al., 2013). Finally, *FT-like* homologs have been identified as negative regulators of flowering in *Beta vulgaris*, *Glycine max*, *Helianthus annuus*, *Nicotiana tabacum*, and *Populus* spp., a function more similar to the canonical role of *TFL1-like* than to most other *FT-like* genes (Blackman et al., 2010; Pin et al., 2010; Hsu et al., 2011; Harig et al., 2012; Wang et al., 2015).

Although *FT*/*TFL1* homologs have been studied in model eudicots and monocots, few studies are available in non-model monocots like the orchids. With approximately 25,000 species, the Orchidaceae is one of the most diversified families of flowering plants. Orchid flowers exhibit predominantly bilateral symmetry as a result of the elaborated medial petal (called the lip) and the congenital fusion of one (rarely 3) stamens with the stigmas into a gynostemium (Pabón-Mora and González, 2008; Hsu et al., 2015). Such floral complexity is translated into exceedingly variable arrays of form, color and size which appeals the market for ornamental plants (Teixeira da Silva, 2013; Teixeira da Silva et al., 2014). Nonetheless, orchid sale and trade faces huge limitations due to their long vegetative phase and intractable indoor flowering seasons (Wang et al., 2017). Little is known about the role of *FT* and *TFL1* homologs during the reproductive transition in orchids (Huang W. et al., 2012; Teixeira da Silva et al., 2014). Isolation and characterization of *FT* homologs have only been done in *Cymbidium* (Huang W. et al., 2012; Xiang et al., 2012), *Dendrobium* (Li et al., 2012; Wang et al., 2017), *Oncidium* (Hou and Yang, 2009), and *Phalaenopsis* (Li et al., 2014; Jang et al., 2015; Zhou et al., 2018), where they appear to positively regulate reproductive transition. For instance, heterologous expression of *FT* orchid homologs in *A. thaliana*, positively regulates *AP1* and promotes early flowering (Hou and Yang, 2009; Huang W. et al., 2012; Li et al., 2012; Zhou et al., 2018). The same results are found when *FT* orchid homologs are transformed into *Oryza sativa* and *Nicotiana tabacum* (Xiang et al., 2012; Jang et al., 2015). The mutant phenotypes of *ft A. thaliana* plants can be partially or completely reverted by the overexpression of orchid *FT* homologs (Hou and Yang, 2009; Li et al., 2014; Jang et al., 2015). Endogenous experiments have only been standardized in *Dendrobium*, where overexpression of *DOFT* (the *FT* homolog) results in early flowering (Wang et al., 2017). Interestingly, *DOFT* is also involved in pseudobulb formation (Wang et al., 2017). In contrast, *TFL1* homologs have been less studied when compared to *FT* homologs. The only functional report shows that *Oncidium TFL1* can delay flowering when overexpressed in wild type *A. thaliana* plants and can fully rescue *tfl1* mutant phenotypes (Hou and Yang, 2009), suggesting conserved roles across *TFL1* homologs.

In this context, our long-term goal is to understand the role of *FT*/*TFL1-like* homologs in the vegetative-to-flowering transition in orchids, particularly in neotropical members with diverse reproductive strategies in natural conditions. Here we present the first large scale analysis of *FT*/*TFL1-like* genes in monocots with an expanded sampling in Orchidaceae. This was done through extensive searches in public repositories as well as ten newly generated orchid transcriptomes from neotropical species. Our analyses point to more duplication events and diversification of *FT* homologs when compared to *TFL1* genes in orchids than previously thought. Finally, we compare protein sequences and expression patterns in three selected species and present hypotheses on the putative role of these genes in their reproductive transition. Our results suggest a possible loss of *TFL1* homologs and a functional shift of some *FT* homologs to repress flowering.

## Materials and Methods

### Reference Transcriptome Sequencing for Neotropical Orchidaceae

In order to identify *FT*/*TFL1-like* genes from neotropical orchids (most with horticultural value), ten transcriptomes corresponding to: *Elleanthus aurantiacus*, *Gomphichis scaposa*, *Masdevallia coccinea*, *M. wendlandiana*, *Maxillaria aurea*, *Miltoniopsis roezlii*, *Oncidium* “Gower Ramsey,” *Oncidium* “Twinkle,” *Stelis pusilla* and *Tolumnia* “Cherry red x Ralph yagi” were sequenced (Supplementary Table S1). The plant material was obtained from individuals grown in nurseries or in the wild in the surroundings of Medellín, Antioquia (Colombia). The reference transcriptomes were sequenced from total RNA extracted from mixed material that included vegetative (with forming leaves) and reproductive meristems (with developing flowers), as well as leaves and pseudobulbs, when present. RNA was isolated using TRIsure (Bioline, London, United Kingdom) following the manufacturer’s instructions, resuspended in ethanol 100% and shipped to the sequencing facilities. The libraries were made using the TruSeq mRNA library construction kit (Illumina, San Diego, CA, United States) and sequenced on a NovaSeq 6000 equipment (Illumina, San Diego, CA, United States) with paired end readings of 100 base pairs. The transcriptome was assembled *de novo* with Trinity V2 at the Centro Nacional de Secuenciación Genómica (CNSG), following the default settings and adding the Trimmomatic adapter removal step. Read cleaning was performed with prinseq-lite v0.20.4 with a quality threshold of Q35 and a minimum read length of 50 bases. Contig metrics are summarized in Table 1.

**TABLE 1:** Assembly statistics of selected orchid species with mixed transcriptomes sequenced.

**Species**	**Total length of sequence (bp)**	**Total number of sequences**	**Average contig length (bp)**	**Largest contig (bp)**	**N50 stats (bp)**	**GC%**
*Elleanthus aurantiacus*	100537418	91814	1095	12295	1800	43.47
*Gomphichis scaposa*	85109234	78810	1079	18109	1748	44.41
*Masdevalia coccinea*	102138335	124799	818	15492	1454	41.11
*Masdevalia wendlandiana*	78531621	75953	1033	15559	1682	41.50
*Maxilaria aurea*	72925370	68647	1062	12136	1700	44.59
*Miltoniopsis roezlii*	64965610	59091	1099	11852	1755	43.48
*Oncidium “*Gower Ramsey”	69431269	84942	817	11821	1176	44.57
*Oncidium “*Twinkle”	74124885	75431	982	11607	1557	43.04
*Stelis pusilla*	90879205	109206	832	11960	1326	43.47
*Tolumnia “*Cherry red *x* Ralph yagi”	94692300	95180	994	12362	1586	42.07

### Isolation of *FT*/*TFL1-Like* Homologs

In order to evaluate the evolution of PEBP genes we generated a comprehensive sampling starting within Orchidaceae and then expanding into other monocots for an inclusive phylogenetic context. In order to isolate *FT*/*TFL1-like* homologs from Orchidaceae, searches were made using BLAST (Altschul et al., 1990) on our orchid transcriptomes as well as on public repositories available. Queries used included the *MFT*/*FT*/*TFL1* reported genes for *Arabidopsis thaliana* (Bradley et al., 1997; Kobayashi et al., 1999) and *Oncidium* Gower Ramsey (Hou and Yang, 2009). Repositories used for Orchidaceae included Orchidbase 3.0 (Fu et al., 2011)^1^ and Orchidstra 2.0 (Chao et al., 2017)^2^. Additionally, some of the *FT*/*TFL1* genes previously reported in other orchids (Hou and Yang, 2009; Huang W. et al., 2012; Xiang et al., 2012; Li et al., 2014; Jang et al., 2015; Wang et al., 2017; Zhou et al., 2018) and monocots outside of the Orchidaceae, using databases like the Rice Genome Annotation Project web^3^, were included (Danilevskaya et al., 2008; Lee et al., 2013; Chaurasia et al., 2017; Leeggangers et al., 2018).

In addition to monocot genes we included the canonical *Arabidopsis* genes for reference and sampled representative taxa from each major angiosperm lineage namely, rosids, asterids, basal eudicots, and basal angiosperms/magnoliids. For additional sampling across angiosperms, we searched NCBI^4^, OneKP^5^, Phytometasyn^6^, Phytozome^7^, and included homologs reported by Pin et al. (2010); Hsu et al. (2011), Navarro et al. (2011), and Harig et al. (2012). We also searched for *FT*/*TFL1-like* homologs in transcriptomes available in our lab for the Magnoliids *Aristolochia fimbriata* and *A. ringens*, the monocots *Cattleya trianae* and *Hypoxis decumbens*, and the eudicots *Bocconia frutescens* (Papaveraceae), *Brunfelsia australis*, and *Streptosolen jamesonii* (Solanaceae) (Pabón-Mora et al., 2015; Arango-Ocampo et al., 2016; Madrigal et al., 2017; Ortiz-Ramírez et al., 2018; Suárez-Baron et al., 2019).

### Phylogenetic Analyses

All the isolated sequences were compiled in Bioedit^8^ and cleaned manually to find the ORF and keep exclusively the CDS of all hits. The nucleotide sequences were aligned using the online version of MAFFT^9^ with a gap opening penalty of 3.0, an offset value of 1.0 and all other default criteria. The alignment was manually edited using the CDS and ORF. The data matrix in PHYLIP format was used for phylogenetic analyses by Maximum Likelihood (ML) using the IQ-TREE software (Nguyen et al., 2015)^10^. The molecular evolution model that best fit the data was calculated using the ModelFinder tool incorporated in IQ-TREE (Kalyaanamoorthy et al., 2017). The Ultrafast Bootstrap (UFBS) of 1000 pseudo-replicas also implemented in IQ-TREE was used to calculate branch support (Hoang et al., 2018). One of the *MFT* sequences of *Amborella trichopoda* (*AmtrMFT1*) was used as outgroup. This particular sequence has the average size for other PEBP genes (ca. 525 bp). The trees were visualized and edited in FigTree v1.4.3^11^. In addition to the complete analysis of *FT*/*TFL1-like* homologs, independent ML analyses were performed for the *FT-like* and *TFL1-like* clades, using *A. trichopoda AmtrTFL1* and *AmtrFT1* as outgroups, respectively.

### Protein Sequence Analysis

In order to identify new protein motifs, as well as those previously reported for the FT/TFL1-like proteins, permanently translated sequences were introduced to the MEME online server with default parameters (Bailey et al., 2009)^12^. We included the canonical FT/TFL1 homologs from *A. thaliana*, selected eudicot and monocot FT/TFL1 genes reported in the literature, as well as the FT/TFL1-like homologs included in our RT-PCR expression analyses (Bradley et al., 1997; Kobayashi et al., 1999; Yoo et al., 2004, 2010; Jang et al., 2009; Pin et al., 2010; Hsu et al., 2011; Navarro et al., 2011; Harig et al., 2012; Huang N. C. et al., 2012; Lee et al., 2013; Zhou et al., 2018). Additionally, specific aminoacids have been reported as crucial for promoting or repressive functioning for FT/TFL1-like proteins in *A. thaliana* (Hanzawa et al., 2005; Ahn et al., 2006; Ho and Weigel, 2014). In order to test whether these positions are conserved in Orchidaceae, an alignment with MAFFT was performed on the TranslatorX web server (Abascal et al., 2010)^13^. Sequences analyzed include FT-like monocot sequences, the canonical sequences of *A. thaliana* (Bradley et al., 1997; Kobayashi et al., 1999; Jang et al., 2009; Yoo et al., 2010, Huang N. C. et al., 2012) and some FT-like copies with repressive function from *Beta vulgaris*, *Nicotiana tabacum*, and *Populus trichocarpa* (Pin et al., 2010; Hsu et al., 2011; Harig et al., 2012).

### RT-PCR and qRT-PCR Expression Analyses

To analyze and compare the expression patterns of the *FT*/*TFL1-like* homologs identified from the newly generated Orchidaceae transcriptomes, three species were selected: *Cattleya trianae*, *Elleanthus aurantiacus*, and *Gomphichis scaposa*. These three species were selected because they had a low copy number of *FT* and *TFL1* gene clades. In addition, they represent different subfamilies as well as diverse habits. Dissections of vegetative (SAM) and inflorescence (IM) meristems, flower buds (FB), leaves (L), axillary buds (AB), and pseudobulbs (PS, present only in *C. trianae*) were made for each species and were flash frozen in liquid nitrogen. Vegetative meristems were taken from plants exclusively producing leaves after all surrounding old and young leaves were removed. Only young leaves were collected separately. Inflorescence meristems (IM) were dissected from enlarged and thickened apices from which all visible young FBs, if present, were removed. All FBs (i.e., those that could be detected by eye) were pulled together and processed as a single sample. Axillary buds were only sampled from *C. trianae*, and they correspond to buds that are still vegetative but have the potential to form new inflorescences in the axil of the bracts.

Total RNA was extracted from each dissected tissue using TRIsure (Bioline, London, United Kingdom) as explained above and it was resuspended in 20 μl of autoclaved miliQ water. The RNA was treated with DNAseI (Invitrogen, Waltham, MA, United States) and quantified with NanoDrop 2000 (Thermo Fisher Scientific, Waltham, MA, United States). A total of 3.0 μg of RNA were used as a template for cDNA synthesis using SuperScript III RT (Invitrogen, Waltham, MA, United States). For the amplification of *FT*/*TFL1-like* genes, specific primers were designed for each copy avoiding conserved domains and sometimes using the UTRs (Supplementary Table S2). Each amplification reaction incorporated 9.0 μl of EconoTaq (Lucigen, Middleton, United States), 5.3 μl of nuclease-free water, 1.0 μl of BSA (5 μg/ml), 1.0 μl of Q solution (5 μg/ml), 1.3 μl of forward primer (10 mM), 1.3 μl of reverse primer (10 mM), 0.1 μl of MgCl2 and 1.0 μl of template cDNA, for a total of 20 μl. The genes were amplified by Touchdown PCR (TD-PCR) (Korbie and Mattick, 2008), following the next thermal profiles: an initial denaturation step (94°C for 40 s), an annealing step (45–54°C for 40 s) and one extension step (72°C for 40 s). Each TD-PCR was carried out in two phases, the initial one began with an annealing temperature (Tm) 8°C above the Tm calculated for the primers used, which then decreased 1°C per cycle until reaching 2°C below of the original Tm, for a total of 10 cycles; and a second amplification phase of 20 cycles, using 2°C below the original Tm. In total, 30 cycles per reaction were performed. Because amplification was still low and very restricted, a second experiment was carried out using 42 cycles, changing the second phase from 20 to 32 cycles. *ACTIN* was used as a positive control. The amplicons were visualized on 1.5% agarose gel with ethidium bromide and digitally photographed using a Whatman Biometra^®^ BioDoc Analyzer.

To validate the expression patterns detected by RT-PCR for *FT*/*TFL1-like* homolgs, qRT-PCR assays were performed in *Cattleya trianae* dissected tissues. The same dissections, as well as protocols for RNA extraction and cDNA synthesis described above were used. However, 2 μg of RNA were used as a template for cDNA synthesis. The qPCR master mix was prepared using Maxima SYBRGreen/ROX qPCR Master Mix K0222 (Waltham, MA, United States). cDNA was diluted 1:2. Three technical replicates were performed. Specific primers were designed for qRT-PCR (Supplementary Table S2). The thermal cycling regime consisted of one initial step at 95°C for 3 min, then 55 cycles at 95°C for 5 s, 54°C for 5 s, and finally 72°C for 20 s in a qTOWER3 G Real-Time Thermocycler (Analytik Jena, Jena, Germany). Endogenous genes tested included *ACTIN*, *GADPH* and *18S*. Transcript levels for *FT*/*TFL1-like* genes were calculated implementing the 2^–ΔΔCt^ function using *18s* as the endogenous control (Livak and Schmittgen, 2001).

### Scanning Electron Microscopy

SAM, IM, and FBs from *E. aurantiacus* and *G. scaposa* were collected in 70% ethanol and stored for 1 month or longer. Apices and buds were dissected in ethanol 90%. The dissected samples were dehydrated in a progressive ethanol series. Samples were critical point-dried using a Baltec CPD 030 and coated with pure gold using an Emitech K550 sputter coater. Finally, all samples were examined and photographed at 10 kV on a Zeiss SUPRA 40VP scanning electron microscope.

## Results

### Evolution of PEBP Genes

A comprehensive search of PEBP homologs in databases available resulted in 525 PEBP genes belonging to 101 angiosperms (Supplementary Table S3). Such comprehensive sampling resulted in 24 sequences of 9 Amborellaceae/Nymphales/Austobaileyales (ANA)/Magnoliid species, 147 genes of 25 eudicot species and 354 genes from 67 monocot species. Specifically, monocot sampling included 130 sequences of 15 non-Orchidaceae species and 224 of 52 species belonging to Orchidaceae.

Complete nucleotide sequences of all isolated homologs were used in the Maximum Likelihood (ML) analysis. The aligned matrix had a total of 1784 characters. The *MFT* homolog of *Amborella trichopoda* (*AmtrMFT1*) was used as outgroup. The resulting topology shows a duplication event prior to angiosperm diversification (UFBS = 100), which results in the *FT-like* (UFBS = 100) and *BFT*/*TFL1-like* (UFBS = 100) clades (Supplementary Figure S1). The *MFT-like* copies are not clustered together but rather form the *MFT-like* grade, which predates the *FT*/*TFL1-like* duplication (Supplementary Figure S1). Within this grade *MFT1* and *MFT2-like* groups were labeled (Drabešová et al., 2016). Because of the large number of sequences and the divergence between the *FT* and the *BFT*/*TFL1-like* copies, subsequent independent ML analyses for each clade were performed. For these analyses the *Amborella trichopoda AmtrTFL1* and *AmtrFT1* were used as outgroups in the separate *FT-like* and *BFT*/*TFL1-like* analyses, respectively.

### *FT-Like* Gene Evolution

**FIGURE 1:**
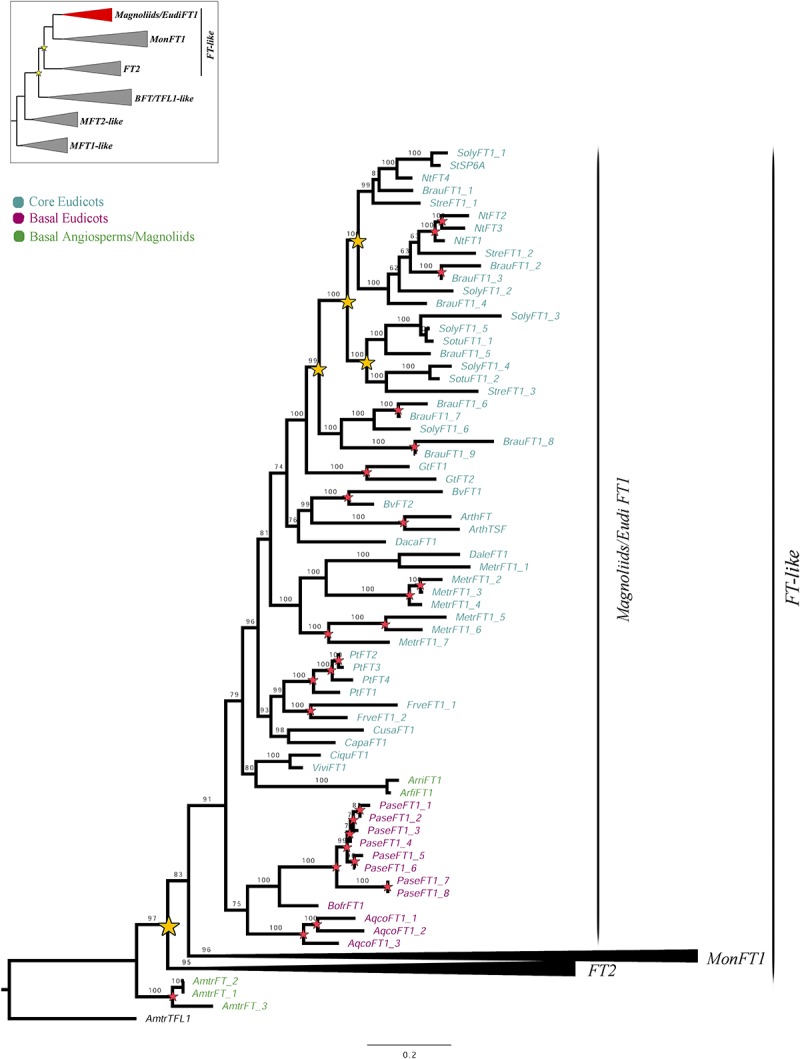
ML analysis of *Magnoliids*/*EudiFT1* genes. Summary tree (upper left), the expanded clade in the figure is indicated in red. Yellow stars indicate large-scale duplication events, while red stars represent intra-specific duplications. Numbers on each node indicate the Ultrafast Bootstrap (UFB) values. The collapsed clades correspond to *MonFT1* (Figure 2) and *FT2* (Figure 3). The colors correspond to the conventions on the left. Scale: 0.2.

**FIGURE 2:**
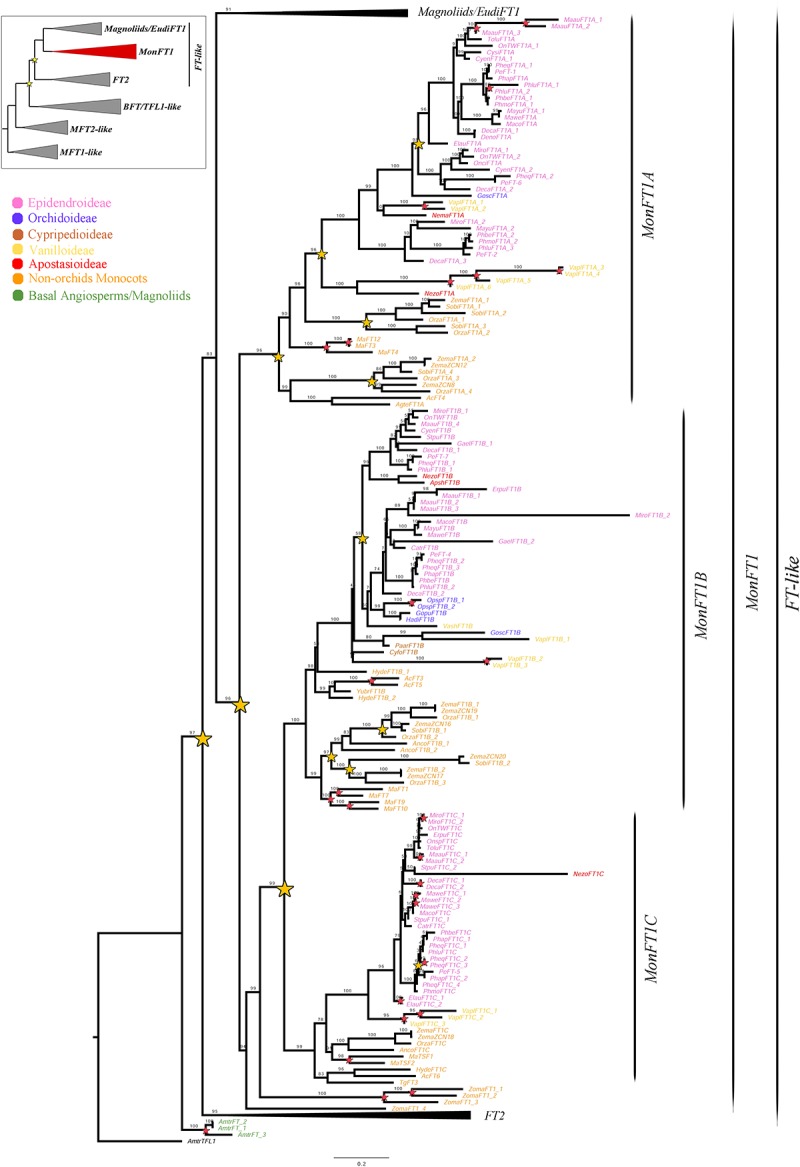
ML analysis of *MonFT1* genes. Summary tree (upper left), the expanded clade in the figure is indicated in red. Star, branch values and color conventions follow those in Figure 1. The collapsed clades correspond to *Magnoliids*/*EudiFT1* (Figure 1) and *FT2* (Figure 3). Scale: 0.2.

**FIGURE 3:**
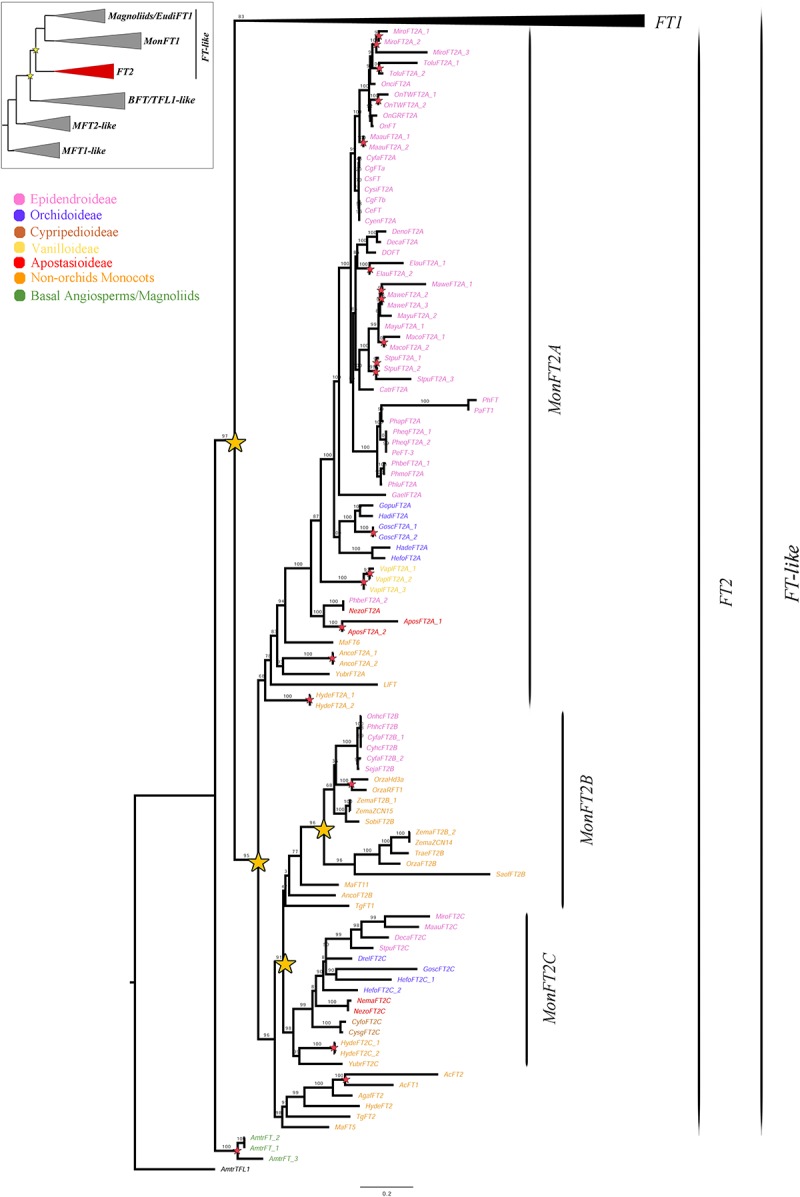
ML analysis of *MonFT2* genes. Summary tree (upper left), the expanded clade in the figure is indicated in red. Stars, branch values and color conventions follow those in Figure 1. The collapsed clade corresponds to *FT1* (Figures 1, 2). Scale: 0.2.

Our sampling includes 81 sequences from 15 non-Orchidaceae monocot species and 192 sequences from 48 Orchidaceae species belonging to different subfamilies, as follows: 146 sequences from 32 species of Epidendroideae, 16 from seven species of Orchidoideae, four from three species of Cypripedioideae, 16 belonging to two species of Vanilloideae and ten of four species of Apostasioideae. Finally, 62 homologs of 19 eudicot species and five of three ANA/Magnoliid species were included. The matrix compiled included a total of 340 sequences and an alignment with 1269 characters. The topology of the ML analysis shows a duplication prior to the diversification of monocots and eudicots, resulting in the *FT1* and *FT2* clades (UFBS = 97) (Figures 1–3). Although there are three *FT* paralogs in *A. trichopoda*, they do not cluster with *FT1* or *FT2*, and appear to be species-specific.

The *FT1* homologs from magnoliids and eudicots form a clade (UFBS = 91) (Figure 1). With our sampling, additional duplications were traced at a family level in Solanaceae. Similarly, local species-specific duplications were found in *Aquilegia coerulea*, *Arabidopsis thaliana*, *Beta vulgaris*, *Brunfelsia australis*, *Fragaria vesca*, *Gentiana triflora*, *Medicago truncatula*, *Nicotiana tabacum*, *Papaver setigerum*, and *Populus trichocarpa*. No *FT2* homologs were recovered from any magnoliid or eudicot species.

Monocot *FT* genes separate into two clades, *MonFT1* and *MonFT2*, coinciding with the duplication described above. The *MonFT1* genes have undergone at least two additional duplications, resulting in the *MonFT1A* (UFBS = 96), *MonFT1B* (UFBS = 100), and *MonFT1C* (UFBS = 99) clades (Figure 2). Within the *MonFT1A* clade, an additional duplication prior to diversification of all monocots was detected, but only one of the resulting copies was retained in orchids. The *MonFT1B* genes have undergone independent duplications in Orchidaceae and Poaceae. Specifically, our analysis identified at least one duplication in orchids and three additional duplications before the diversification of Poaceae in this subclade. Conversely, the *MonFT1C* copy is duplicated only in *Phalaenopsis* (Epidendroideae, Orchidaceae). Species-specific duplications of *MonFT1* have occurred in *Allium cepa*, *Dendrobium catenatum*, *Elleanthus aurantiacus*, *Masdevallia wendlandiana*, *Maxillaria aurea*, *Miltoniopsis roezlii*, *Musa acuminata*, *Ophrys sphegodes*, *Phalaenopsis lueddemanniana*, *P. equestris*, *Vanilla planifolia*, and *Zostera marina*.

As previously explained, the *FT2* genes appear to be exclusive to monocots (Figure 3). Within monocots, these genes were duplicated at least twice (UFBS = 95 and 91 respectively) resulting in the *MonFT2A* (UFBS = 68), *MonFT2B* (UFBS = 42), and *MonFT2C* (UFBS = 98) paralogs. The position of the *Allium cepa AcFT1* and *AcFT2*, the *Agapanthus africanus AgafFT2*, the *Ananas comosus AncoFT2B*, the *Hypoxis decumbens HydeFT2*, the *Musa acuminata MaFT5* and *MaFT11*, and the *Tulipa gesneriana TgFT1* and *TgFT2* homologs supports the timing of the duplication prior to monocot diversification. Additional large-scale duplications were only found in the *MonFT2B* clade, which was duplicated once prior to the diversification of Poaceae and Orchidaceae (UFBS = 96) with the retention of one of the copies in Orchidaceae, specifically in the Epidendroideae subfamily. Finally, species-specific duplications were found in *Allium cepa*, *Ananas comosus*, *Apostasia wallichii*, *Elleanthus aurantiacus*, *Gomphichis scaposa*, *Hypoxis decumbens*, *Masdevallia coccinea*, *Masdevallia wendlandiana*, *Maxillaria aurea*, *Miltoniopsis roezlii*, *Oncidium* “Twinkle,” *Oryza sativa*, *Stelis pusilla*, *Tolumnia* “Cherry red x Ralph yagi” and *Vanilla planifolia*.

### *BFT*/*TFL1-Like* Gene Evolution

**FIGURE 4:**
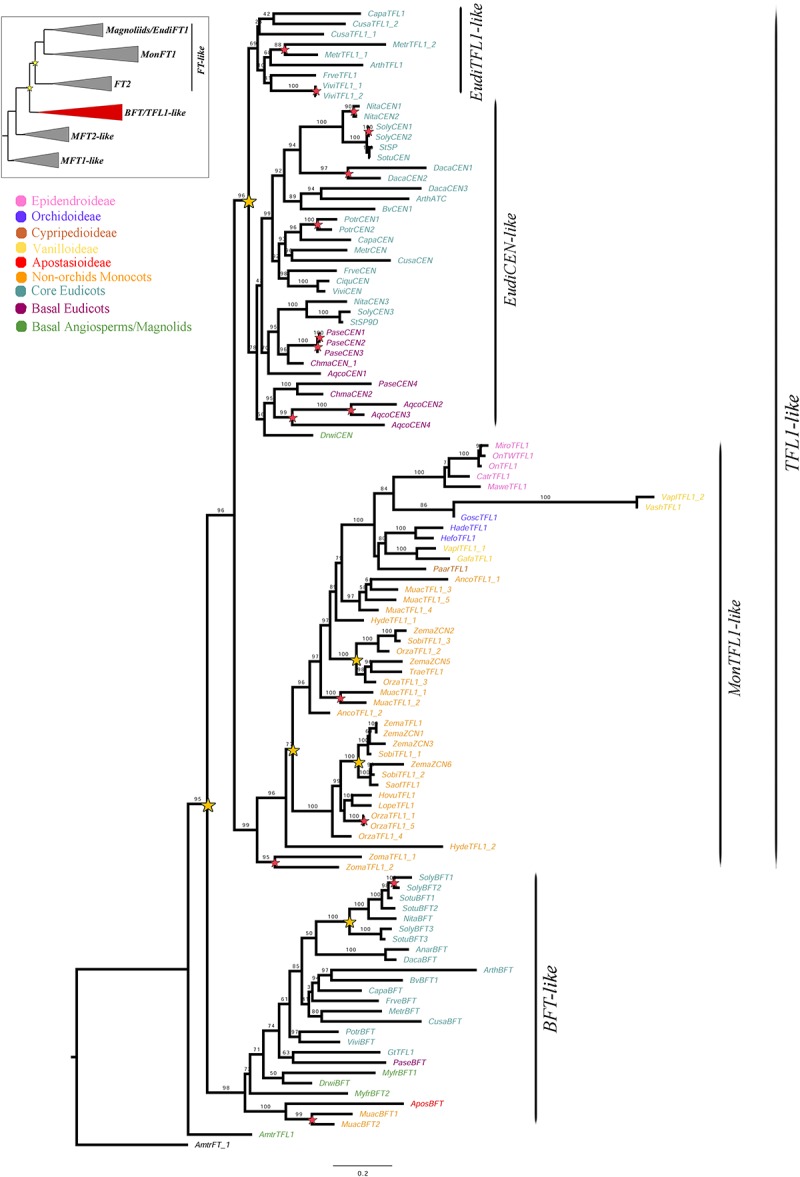
ML analysis of *BFT*/*TFL1-like* genes. Summary tree (upper left), the expanded clade in the figure is indicated in red. Star, branch values and color conventions follow those in Figure 1. Scale: 0.2.

Despite the exhaustive sampling only 110 *BFT*/*TFL1-like* homologs were isolated, which account for one fifth of the total sampling. Our sampling includes five genes of Epidendroideae (5 spp.), three genes of Orchidoideae (3 spp.), one gene of Cypripedioideae (1 sp.), one gene of Apostasioideae (1 sp.), four genes of Vanilloideae (3 spp.), as well as 31 genes of 11 non-Orchidaceae monocots, 60 of 18 eudicots and five from three early divergent angiosperms. The aligned matrix includes 854 characters. ML topology shows a duplication resulting in the *BFT-like* and *TFL1-like* clades (UFBS = 95) (Figure 4). Because the single copy of the *Amborella trichopoda AmtrTFL1* does not nest within either clade, it is likely that the duplication predates the diversification of magnoliids, monocots and eudicots. In addition, a second duplication (UFBS = 96) occurs in eudicots, resulting in the *EudiTFL1-like* and *EudiCEN-like* clades. The *EudiCEN-like* genes are present in all eudicots whereas the *EudiTFL1-like* genes are only found in the super-rosids. This could be explained by two alternative hypotheses: (1) the *EudiTFL1-like* copy was lost in basal eudicots and in Asterids; or (2) the topology may change with expanded sampling, thus the sequences of early divergent eudicots inside *EudiCEN-like* belong in fact to *EudiTFL1-like*, as indicated by the relatively low supports in these branches (UFBS = 78).

Monocot sequences clustered together in a clade here called *MonTFL1-like* (Figure 4), in which an additional duplication can be traced after the divergence of the Alismatales (UFBS = 77). Only one of the copies is subsequently retained in the Orchidaceae. Within Poaceae two additional duplication events can be identified, one prior to the diversification of the family while other specific to the Panicoideae (Figure 4). Finally, although the *BFT* clade has been retained largely as single copy, at least one duplication has occurred in Solanaceae. Species-specific duplications in the *BFT*/*TFL1* clade can be observed in *Aquilegia coerulea*, *Daucus carota*, *Medicago truncatula*, *Musa acuminata*, *Nicotiana tabacum*, *Oryza sativa*, *Papaver setigerum*, *Populus trichocarpa*, *Solanum lycopersicum*, *Vitis vinifera*, and *Zostera marina*.

### Sequence Analysis

In order to identify new protein motifs, as well as those previously reported for PEBP proteins, an analysis was performed with selected sequences on the MEME server. This analysis included the permanently translated sequences from orchid species used in expression analyses (see below) and some of the previously reported sequences with expression or functional data available. We found that motifs 1–6 are present in most of the homologs analyzed, while all other motifs are only found in some proteins (Supplementary Figure S2). For example, motif 7 is exclusive of *Phalaenopsis equestris* PeFT-2 and PeFT-5, motifs 8, 12, and 13 are only present in some sequences belonging to the *MonFT2* clade, motifs 9 and 14 are characteristic of some proteins of *BFT*/*TFL1* clade and motif 10 is shared between *Elleanthus aurantiacus* ElauFT2A1 and PeFT-5. Finally, motif 11 is only present in ArthATC, ArthTFL1, and PeFT-5.

**FIGURE 5:**
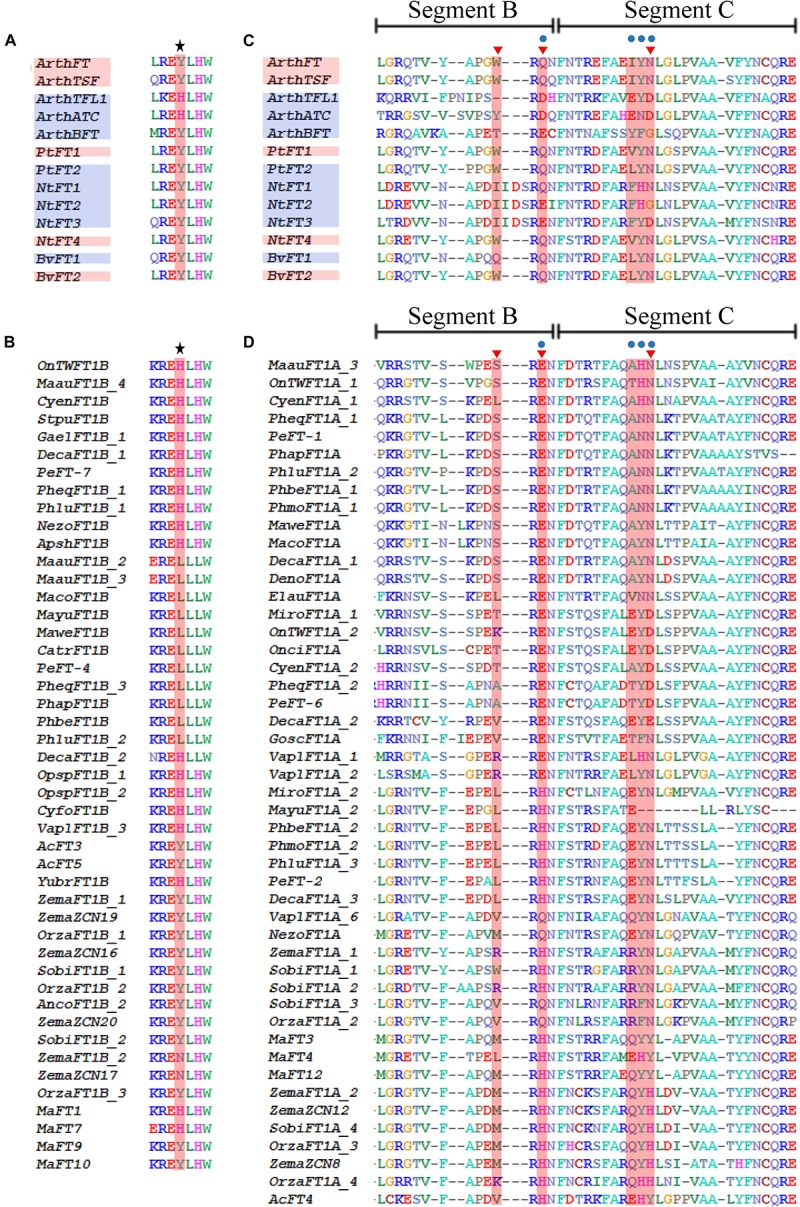
Sequence comparison between MonFT1A, MonFT1B and the canonical PEBP sequences from *Arabidopsis thaliana* and selected FT eudicot sequences with repressive function on key amino acids distinguishing FT and TFL1-like homologues (following Hanzawa et al., 2005; Ahn et al., 2006; Ho and Weigel, 2014). **(A)** Alignment of the positions homologous to Tyr-85 (Y)/His-88 (H) (in red and with upper star) from FT and TFL1 of *A. thaliana* respectively, as well as other repressive PEBP proteins from *Beta vulgaris* (*Bv*), *Nicotiana tabacum* (*Nt*) and *Populus trichocarpa* (*Pt*). **(B)** Same positions in the *MonFT1B* proteins. **(C)** Alignment of segments B and C of selected PEBP homologs, including canonical PEBP as well as FT homologs with repressive function. Blue dots (following Ahn et al., 2006) and red triangles (following critical residues identified by Ho and Weigel, 2014) indicate key amino acids distinguishing FT and TFL1-like homologs. **(D)** Same positions of segments B and C in the *MonFT1A* proteins. Red boxes on the sequence names indicate proteins with flowering promoter function, while the blue boxes indicate proteins with repressive function.

Other aminoacids have been assigned with specific roles for FT and TFL1 proteins. For instance, in *A. thaliana*, the FT Tyr-85 (Y) and the TFL1 His-88 (H) homologous amino acids are important to confer part of the characteristic function of each protein, that is to promote or repress flowering, respectively (Hanzawa et al., 2005). The Tyr-85 and His-88 are located in motif 3 (Supplementary Figure S2). Same positions were evaluated across FT-like protein monocot homologs, and it was found that some of the *MonFT1B* sequences possess the characteristic H from TFL1 instead of the expected Y from FT (Figures 5A,B).

Functional analyses using protein chimeras have also identified that peptide sequence of segments B and C in the FT and TFL1 proteins of *A. thaliana* are responsible for the role of each protein to promote or delay flowering (Ahn et al., 2006). Segments B and C include regions from motifs 4, 5, and 6 (Supplementary Figure S2). The amino acid sequence of segment B is conserved among FT-like proteins, whereas in TFL1-like proteins this segment evolves rapidly and therefore is variable between close homologs (Ahn et al., 2006). When segment B is compared to FT-like homologs isolated from monocots, we find that it is highly conserved in all *MonFT* clades except for the proteins belonging to the *MonFT1A* clade (Figures 5C,D and Supplementary Figure S3). These sequences are highly variable and, thus, they resemble TFL1-like proteins (Figures 5C,D; Ahn et al., 2006). Inside segment B, a residue has been identified in the homologous position Gln-140 (Q) and Asp-144 (D) to distinguish FT from TFL1 in *A. thaliana* respectively (Ahn et al., 2006). Our sequence analysis revealed that in members of the *MonFT1A* clade the Gln (Q) is replaced by Glu (E) or His (H) (Figure 5D).

On the other hand, segment C is very similar between FT and TFL1 proteins. However, a few amino acids located between the homolog positions 150–152 and 154–156 can be used to distinguish between putative FT or TFL1 proteins (Ahn et al., 2006). When monocot FT proteins are compared in these positions, all MonFT proteins except for those belonging to MonFT1A show predictive residues of FT activity (IYN in Figures 5C,D and Supplementary Figure S3). Same positions in MonFT1A are highly variable and cannot be used to predict any functional activity by comparison with the canonical FT or TFL1 (Figures 5C,D).

Finally, between segment B and C, specific mutations in each of four critical residues, Glu-109 (E), Trp-138 (W), Gln-140 (Q), and Asn-152 (N), can turn FT into a TFL1-like floral repressor (Ho and Weigel, 2014). When monocot FT proteins are compared in these positions, all MonFT proteins conserve predictive residues of FT promoting function except MonFT1A (WQN in Figures 5C,D and Supplementary Figure S3). These positions are highly variable in MonFT1A proteins, however, they have aminoacids more similar to TFL1 than to FT (Figures 5C,D).

### Expression Analyses

**FIGURE 6:**
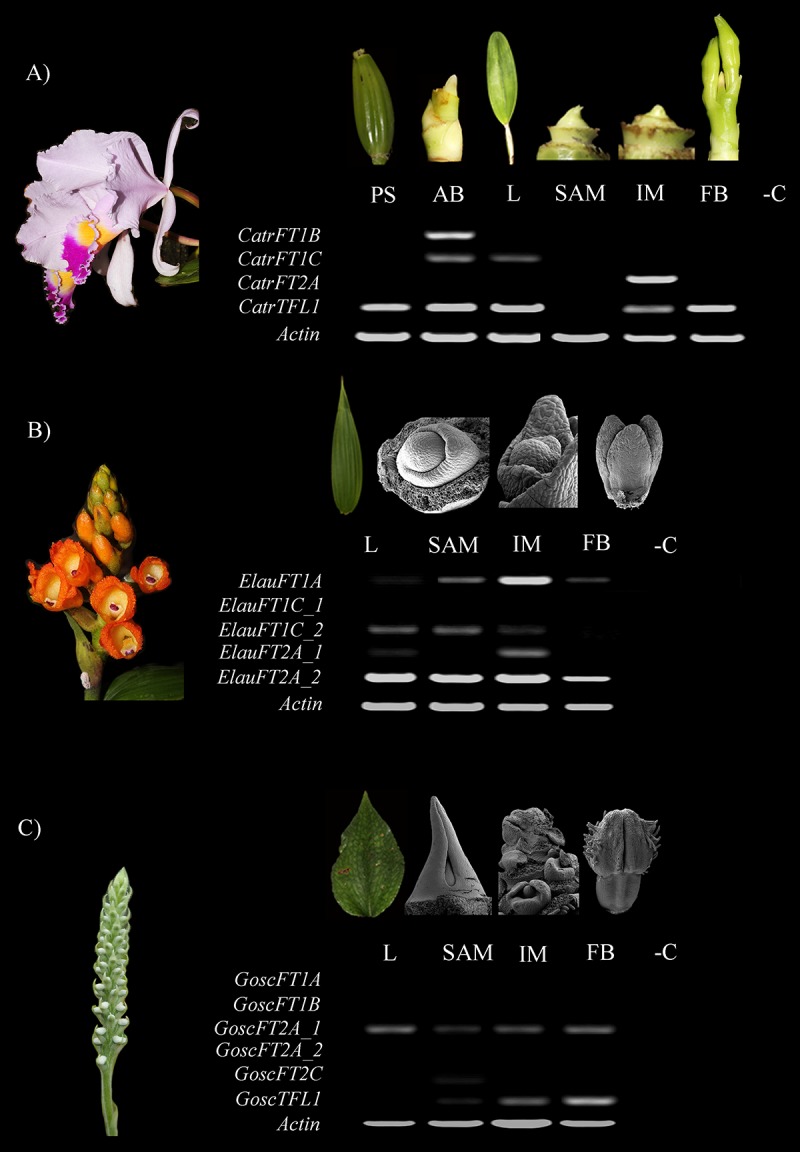
Expression analyses of *FT*/*TFL1-like* genes in selected orchids using TD-PCR and 42 cycles. **(A)** *Cattleya trianae*. **(B)** *Elleanthus aurantiacus*. **(C)** *Gomphichis scaposa*. AB, Axillary bud; BF, Floral bud; IM, Inflorescence meristem; L, Leaf; PB, Pseudobulb; SAM, Apical vegetative meristem. Each dissection in *C. trianae* corresponds with a light stereoscope photograph, together with the *E. aurantiacus* and *G. scaposa* leaves. All other photographs correspond to Scanning Electron Microscopy images. -C indicated the PCR amplification lacking cDNA as a negative control.

**FIGURE 7:**
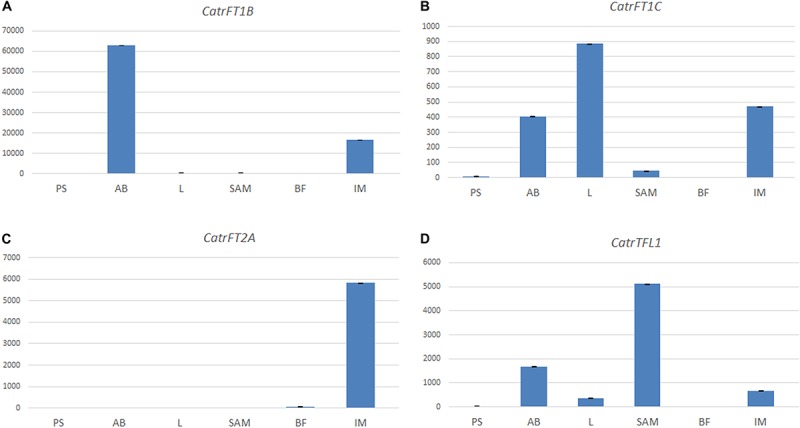
Expression analyses of *FT*/*TFL1-like* genes in *Cattleya trianae* using qRT-PCR of **(A)** *CatrFT1B*, **(B)** *CatrFT1C*, **(C)** *CatrFT2A*, and **(D)** *CatrTFL1*. *18S* was used as the endogenous control.

In order to observe the expression patterns of the *FT*/*TFL1-like* genes previously identified, we chose three orchid species: *Cattleya trianae*, *Elleanthus aurantiacus* and *Gomphichis scaposa*. These species were selected based on their low copy number of both *FT* and *TFL1* genes. In addition, they represent two different orchid lineages, the Orchidoideae (*G. scaposa*) and the Epidendroideae (*C. trianae* and *E. aurantiacus*). Plants growing in nurseries (*C. trianae*) or in the wild (*E. aurantiacus* and *G. scaposa*) were dissected into leaves (L), vegetative meristem (SAM), inflorescence meristem (IM), and FB. In addition, for *C. trianae* expression was also evaluated in the pseudobulb (PS) and the axillary buds (AB). Amplifications by TD-PCR (Korbie and Mattick, 2008) at 30 cycles and 42 cycles were performed to evaluate minimal and maximal expression level during the vegetative-to-reproductive transition (Figure 6 and Supplementary Figure S4). In addition, qRT-PCR was done to verify RT-PCR results in a quantitative manner for *C. trianae* (Figure 7). However, expression analyses results presented here describe the 42 cycle RT-PCR and the qRT-PCR when available.

In *Cattleya trianae* RT-PCR allowed us to identify expression of *CatrFT1B* restricted to leaves (L), expression of *CatrFT1C* in axillary buds (AB) and leaves (L), expression of *CatrFT2A* in the IM and broad expression of *CatrTFL1* in PS, AB, L, IM, and FB (Figure 6A). qRT-PCR expression validates the RT-PCR analyses and in addition records the expression of *CatrFT1B* and *CatrFT1C* in IM (Figure 7).

RT-PCR in *Elleanthus aurantiacus* identifies the expression of *ElauFT1A* in all tissues dissected, especially in SAM and IM. Furthermore, *ElauFT1C_2* and *ElauFT2A_1* expression is detected in L, SAM and the IM, while *ElauFT1A_2* is broadly expressed in L, SAM, IM, and FB (Figure 6B).

Finally, RT-PCR in *Gomphichis scaposa* results in the detection of *GoscFT2A_1* in L, SAM, IM and BF, expression of *GoscTFL1* in the SAM, the IM and the FB, and very low expression of *GoscFT2C* in the SAM. No expression is detected for *GoscFT1A*, *GosFT1B*, or *GoscFT2A_2* (Figure 6C).

## Discussion

Previous phylogenetic analyses have divided the *PEBP* gene family in plants into the *MFT-like*, *FT-like* and *TFL1-like* subfamilies (Chardon and Damerval, 2005; Carmona et al., 2007; Danilevskaya et al., 2008; Hedman et al., 2009; Karlgren et al., 2011; Liu et al., 2016). Our ML results recover the same three groups, where the *MFT-like* grade is sister to the *BFT*/*TFL1-like* and *FT-like* clades (Supplementary Figure S1 and Figures 1–4). Monophyly and evolution of the *MFT-like* genes have been extensively debated (Hedman et al., 2009; Liu et al., 2016). In that respect, our analyses recover most *MFT* homologs from magnoliids, monocots and eudicots group with the canonical *A. thaliana MFT*. However, some *MFT* genes from few early divergent angiosperms and eudicots form a different group in the absence of *A. thaliana* homologs. It is likely that these genes belong to yet another subfamily that has been comparatively less sampled (Drabešová et al., 2016). Within the *FT*/*TFL1-like* clades we were able to identify a number of large scale as well as local duplications and the associated changes in protein sequences in the resulting paralogs. Some had been previously identified (Chardon and Damerval, 2005; Carmona et al., 2007; Danilevskaya et al., 2008; Hedman et al., 2009; Karlgren et al., 2011; Lee et al., 2013; Liu et al., 2016; Chaurasia et al., 2017; Leeggangers et al., 2018) while some are reported here for the first time.

### The *FT-Like* Gene Subfamily Has Undergone Reiterative Duplication Events Compared to the *TFL1-Like* Subfamily in Monocots

From the three PEBP subfamilies, the *FT-like* genes are by far the most diversified in terms of copy number (Chardon and Damerval, 2005; Danilevskaya et al., 2008; Lee et al., 2013; Chaurasia et al., 2017). Our ML analyses suggest that *FT-like* homologs have undergone a duplication event prior to the diversification of monocots and eudicots, giving rise to copies *FT1* and *FT2* (Figures 1–3). While *FT1* genes, the *A. thaliana FT* orthologs, are widespread across angiosperms (Figures 1, 2), *FT2* genes are retained exclusively in monocots (Figure 3). Monocot *FT1* and *FT2* genes have also duplicated extensively. We were able to recover two duplications in *FT1* genes, resulting in the *MonFT1A*, *MonFT1B*, and *MonFT1C* paralogs, as well as two additional duplications in the *FT2* genes, resulting in the *MonFT2A*, *MonFT2B*, and *MonFT2C* copies. These duplications coincide with whole genome duplication events that occurred during the evolution of monocots (Murat et al., 2017).

### Within *FT-Like* Genes the *MonFT2* Homologs Are Likely Maintaining Their Roles as Flowering Promoters in Orchidaceae

Orchidaceae species sampled have *FT* representatives in all six gene clades, in agreement with the five *FT-like* homologs reported for *Phalaenopsis* (Zhou et al., 2018). Most of the previously reported *FT-like* homologs in the family functionally analyzed (Hou and Yang, 2009; Huang W. et al., 2012; Li et al., 2012; Xiang et al., 2012; Li et al., 2014; Jang et al., 2015; Wang et al., 2017) nest in the *MonFT2A* clade in our analysis. Expression analyses of *FT-like* homologs from the *FT2* clade have identified active transcription of *MonFT2* members in leaves, SAM, IM, FBs, and fruits. These patterns are consistently found in Asparagales (*Allium cepa*), Liliales (*Lilium longiflorum* and *Tulipa gesneriana*), Orchidaceae (*Cymbidium goeringii*, *Dendrobium* “Chao Praya Smile,” *Dendrobium nobile*, *Oncidium* Gower Ramsey, *Phalaenopsis aphrodite*, *Phalaenopsis* “Thailin Red Angel V31,” *Phalaenopsis* “Fortune Saltzman”), Poales (*Zea mays*) and Zingiberales (*Musa acuminata*) (Danilevskaya et al., 2008; Hou and Yang, 2009; Li et al., 2012, 2014; Xiang et al., 2012; Lee et al., 2013; Jang et al., 2015; Manoharan et al., 2016; Chaurasia et al., 2017; Wang et al., 2017; Leeggangers et al., 2018; Zhou et al., 2018). In addition, *FT2* homologs from *A. cepa* and *Oncidium* “Gower Ramsey” have also been detected in bulbs and pseudobulbs, respectively (Hou and Yang, 2009; Lee et al., 2013; Manoharan et al., 2016).

Functional analyses of *MonFT2* representatives have uncovered their role in promoting flowering either endogenously (Kojima et al., 2002; Tamaki et al., 2007; Wang et al., 2017; Leeggangers et al., 2018), or by heterologous transformation in *Arabidopsis thaliana*, *Nicotiana tabacum*, or *Oryza sativa* (Hou and Yang, 2009; Huang W. et al., 2012; Xiang et al., 2012; Li et al., 2014; Jang et al., 2015; Leeggangers et al., 2018; Zhou et al., 2018). In addition, *MonFT2* genes have been shown to induce bulb formation in *Allium cepa* (Lee et al., 2013). A single case has linked *MonFT2* homologs with the negative regulation of flowering after the *Tulipa gesneriana TgFT1* heterologous expression in *A. thaliana* (Leeggangers et al., 2018). In general, most *MonFT2* transcripts are likely the functional homologs of the *A. thaliana FT* triggering vegetative-to-reproductive transition.

Our expression analyses shows that orchid *MonFT2* orthologs are highly expressed compared to all other isolated *FT* homologs. Interestingly, in the two terrestrial orchid species *Elleanthus aurantiacus* and *Gomphichis scaposa* at least one of the two *MonFT2* homologs is broadly expressed in leaves, IM and FBs, while in the epiphytic *Cattleya trianae*, *CatrFT2A* is exclusively turned on in the IM. A detailed comparison of the orchid MonFT2 protein sequences (Supplementary Figure S3) with the canonical promoters and repressors in model core eudicots, shows that they are more likely to function as flowering promoters, like the homologs functionally evaluated in the same clade. Altogether, our data suggests that despite subtle differences in early expression in leaves, SAM and FBs among species with different habits, the role of *MonFT2* homologs as positive flowering regulators seems to be retained in all orchids studied so far.

### Most *MonFT1* Homologs in Orchidaceae Are Likely Repressing Flowering Transition With the Exception of *MonFT1C* Orthologs

The expression of *MonFT1* genes (i.e., those that nest in the *MonFT1* clade in our analysis) has been less studied in orchids when compared to *MonFT2* genes. *MonFT1* transcripts have been isolated from leaves, IM as well as young and old FBs in *Allium cepa* (Asparagaceace), *Tulipa gesneriana* (Liliaceae), *Phalaenopsis* Tailin Red Angel V31 (Orchidaceae), *Zea mays* (Poaceae), and *Musa acuminata* (Musaceae) (Danilevskaya et al., 2008; Lee et al., 2013; Manoharan et al., 2016; Chaurasia et al., 2017; Leeggangers et al., 2018; Zhou et al., 2018). Only rarely they have been isolated from SAMs, stems and roots in *T. gesneriana* and *Z. mays* (Danilevskaya et al., 2008; Leeggangers et al., 2018), as well as from the bulb of *A. cepa* (Lee et al., 2013; Manoharan et al., 2016).

Functional data for *MonFT1* are scarce when compared to data available for *MonFT2* homologs. When overexpressed endogenously in *Z. mays* (Meng et al., 2011) or in a heterologous manner in *A. thaliana*, *MonFT1* genes promote flowering (Lazakis et al., 2011; Chaurasia et al., 2017). However, additional experiments overexpressing the *MonFT1 T. gesneriana* homolog in *A. thaliana* have resulted in a slight delay in flowering (Leeggangers et al., 2018). Conversely, the overexpression of the *M. acuminata MonFT1* homologs in *A. thaliana* has no effect on flowering time (Chaurasia et al., 2017). Finally, *MonFT1* homologs can also regulate bulb formation in *A. cepa* (Lee et al., 2013; Rashid et al., 2019).

Our expression analyses show that *MonFT1* orchid orthologs have a more restricted expression when compared to *MonFT2* homologs, and sometimes, like in the case of *Gomphichis scaposa* expression is lacking from all the dissected organs. *MonFT1* homologs from *Cattleya trianae* and *Elleanthus aurantiacus* can be detected in axillary buds, SAMs and IMs. Only *MonFT1C* homologs can be detected in leaves. Comparison of protein sequences among MonFT1 proteins with the canonical promoters and repressors in core eudicots points to *MonFT1A* and *MonFT1B* as negative regulators of the flowering transition, while *MonFT1C* more likely retain the role of promoting flowering. Thus, *MonFT1A* and *MonFT1B* might have been co-opted for the typical *TFL1-like* roles by delaying flowering and promoting inflorescence indeterminate meristem identity at least in Epidendroideae. This is particularly relevant as *TFL1* homologs are not as abundant as *FT* genes in monocots (see below). From the *MonFT1* genes, only *MonFT1C* genes are more likely functioning as flowering promoters in orchids as they accumulate in leaves and SAMs and IM, similar to what happens with the canonical *FT* from *A. thaliana* (Corbesier et al., 2007).

### *TFL1-Like* Genes Are Completely Lacking or Found in Low Number in Monocots When Compared to Eudicots

Opposite to *FT* genes, the *TFL1* subfamily has diversified more in eudicots than in monocots. Our analyses show an early duplication in angiosperms resulting in the *TFL1-like* and the *BFT-like* clades, in agreement with previous studies (Carmona et al., 2007; Liu et al., 2016). The *BFT-like* genes are present in most angiosperms and they are here proposed as an additional subfamily within the PEBP gene family. However, monocot representatives within this group are scarce suggesting they are likely undergoing pseudogenization. Our results also recover a eudicot specific duplication resulting in the *EudiCEN-like* and *EudiTFL1-like* copies (Figure 4). All monocot *TFL1-like* homologs are clustered in the *MonTFL1-like* clade (Figure 4) consistent to what has been previously reported (Liu et al., 2016; Gao et al., 2017).

Isolation of *TFL1-like* homologs from orchids has proven difficult and so far, only one homolog from *Oncidium* “Gower Ramsey” has been reported (Hou and Yang, 2009). Despite our sampling effort, *TFL1-like* homologs were found only in 12 orchid species, frequently as single copy with the exception of *Vanilla planifolia*, a species with two copies (Figure 4). No *TFL1-like* homologs were found in the genome of *Phalaenopsis equestris* (Cai et al., 2015). The lack of *TFL1-like* homologs in several orchid species may be either the result of the lack of expression of these genes on the sampled tissues, or a gradual loss-of-function of *TFL1-like* genes together with functional compensation by *FT-like* copies, specifically from the *MonFT1A* and *MonFT1B* clades (see above). In the latter scenario, it is possible that monocot specific *FT-like* copies have co-opted novel functions including flowering repression. However, functional analyses are necessary to test this hypothesis.

### The Remaining *MonTFL1* Genes Likely Repress Flowering in Orchidaceae

Some expression analyses have been performed for *MonTFL1-like* homologs (i.e., those that nest in the *MonTFL1* clade in our analysis) in Poaceae (*Lolium perenne*, *Oryza sativa*, *Saccharum* spp., and *Zea mays*). These genes are expressed in leaves, stems, roots, SAMs, IMs, and FBs (Jensen et al., 2001; Zhang et al., 2005; Danilevskaya et al., 2008; Coelho et al., 2014). *MonTFL1-like* genes in orchids have only been characterized in *Oncidium* “Gower Ramsey,” where *TFL1* is expressed in pseudobulbs and axillary buds (Hou and Yang, 2009). The overexpression of *MonTFL1-like* homologs in *Z. mays* delays flowering time and increases lateral branching and spikelet density in the inflorescence (Danilevskaya et al., 2010). Also, *Oncidium MonTFL1-like* can rescue the late flowering and the terminal flower in *tfl1* mutants from *A. thaliana*. Altogether, available data indicate that *TFL1* monocot genes function similarly to their homologs in *A. thaliana*.

Our expression analyses show broad expression of *TFL1-like* homologs in all dissected organs from the epiphytic *Cattleya trianae*. On the other hand, in the terrestrial, understory specialist *Gomphichis scaposa*, *TFL1-like* genes are mainly found in the IM and the FBs. Finally, *Elleanthus aurantiacus*, a terrestrial species growing in full light lacks a *MonTFL1* homolog. Thus, it is possible that copy number of *MonTFL1* genes as well as their expression patterns vary in response to specific habitats. Altogether, evolution and expression patterns point to *MonTFL1* orchid homologs as involved in flowering repression similar to the reports on *TFL1* homolog from *Oncidium* “Gower Ramsey” (Hou and Yang, 2009).

## Conclusion

*FT-like* genes are by far more diversified than *TFL1-like* genes in monocots, reaching up to six clades in the former when compared to a single lineage in the latter. Within *MonFT1* genes, the comparative protein sequences of MonFT1A and MonFT1B suggest that they could have acquired functional roles in delaying flowering, a role assigned so far to TFL1-like proteins. This is further supported by the lack of expression from IMs seemingly uncoupled to the vegetative-to-reproductive transition. On the other hand, *MonFT2* genes have retained their canonical motifs and roles in promoting flowering transition, with increasing expression levels from SAMs and leaves to IMs and FBs. Finally, *TFL1-like* genes are retained as single copy in orchids and can be even lacking from representative genomes (*Phalaenopsis equestris*) and transcriptomes sampled alike. Thus, they are likely undergoing selection toward pseudogenization. Their function could be linked to the parallel recruitment of *MonFT1A* and *MonFT1B* homologs in delaying flowering and maintaining indeterminacy in the inflorescence meristem. Our work also highlights the importance of large-scale genome and transcriptome analyses to build up a comprehensive framework for all *FT*/*TFL1* monocot homologs simultaneously without focusing on specific gene clades. All functional hypotheses postulated here wait for functional validation in emerging model orchid species and comparative analyses in orchids with horticultural value.

## Data Availability Statement

The datasets generated for this study can be found in the NCBI GenBank accession MN968819–MN968822, MN968823–MN968828, MN968829–MN968836, MN968837–MN968849, MN968850–MN968854, MN968855–MN968863, MN968864–MN968875, MN968876–MN968886, MN968887–MN968888, MN968889–MN968890, MN968891–MN968897, MN968898–MN968901, MN968902–MN968907, MN968908–MN968909, MN968910–MN968911, MN968912–MN968922, and MN968923–MN968926.

## Author Contributions

DO-Z, YM, and NP-M planned and designed the research, conducted fieldwork and performed the experiments. JA assembled the reference transcriptomes. All authors analyzed the data, wrote and approved the final manuscript.

## Conflict of Interest

The authors declare that the research was conducted in the absence of any commercial or financial relationships that could be construed as a potential conflict of interest.

## Footnotes

1 http://orchidbase.itps.ncku.edu.tw/est/home2012.aspx

2 http://orchidstra2.abrc.sinica.edu.tw/orchidstra2/index.php

3 http://rice.plantbiology.msu.edu/

4 https://www.ncbi.nlm.nih.gov/genbank/

5 https://db.cngb.org/onekp/

6 https://bioinformatics.tugraz.at/phytometasyn/

7 https://phytozome.jgi.doe.gov/pz/portal.html

8 https://bioedit.software.informer.com/versions/

9 https://mafft.cbrc.jp/alignment/server/

10 http://www.iqtree.org/

11 http://tree.bio.ed.ac.uk/software/figtree/

12 http://meme-suite.org/tools/meme

13 http://translatorx.co.uk/
