# Supplementary material for: Evolution and Expression of Reproductive Transition Regulatory Genes FT/TFL1 With Emphasis in Selected Neotropical Orchids
Source: Front Plant Sci. 2020 Apr 21;11:469. doi: 10.3389/fpls.2020.00469 (PMC7186885; doi:10.3389/fpls.2020.00469)
Supplement: Supplementary file 1 [file Data_Sheet_1.PDF]

Supplementary Material

Supplementary Figures

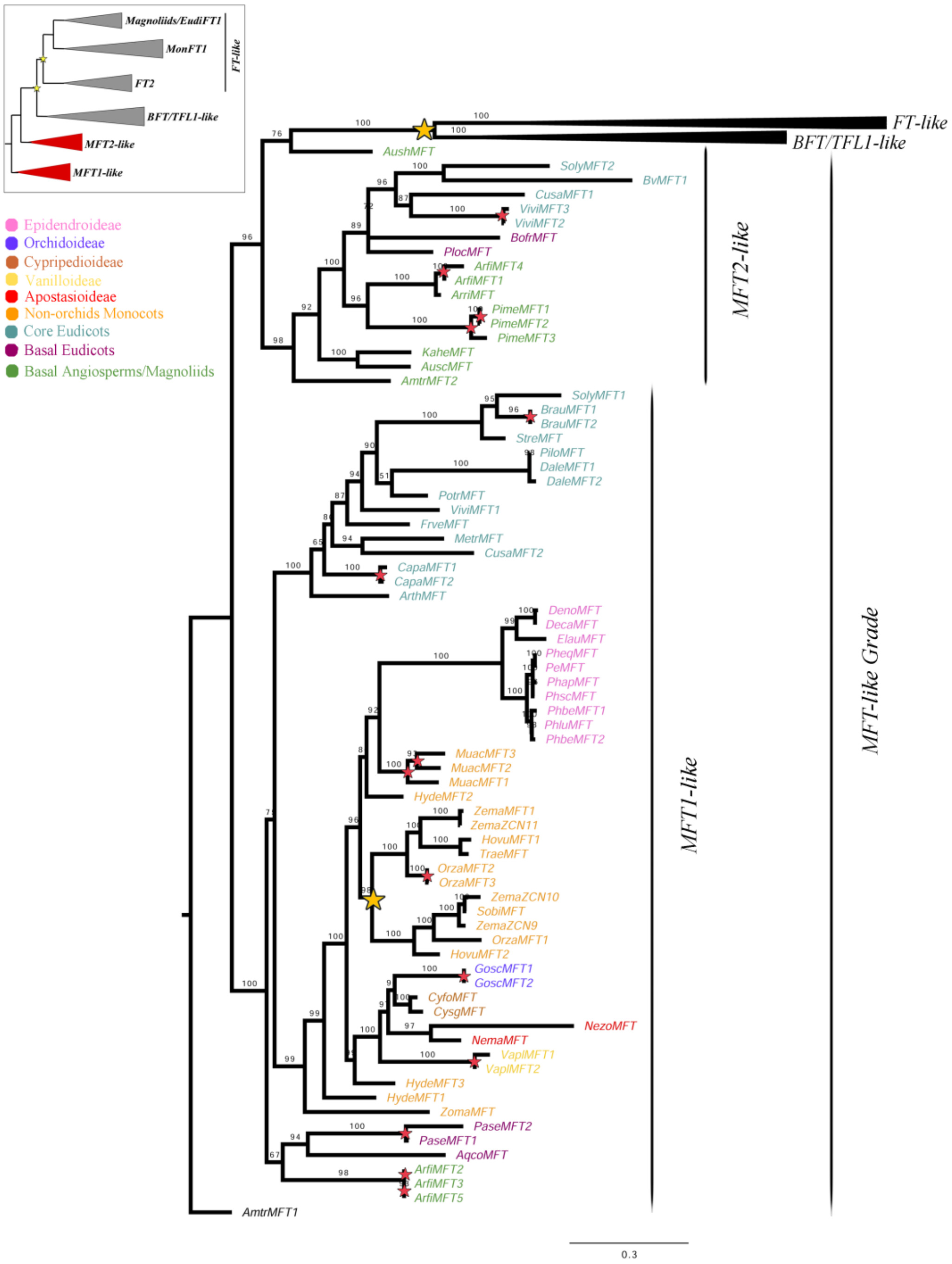

Supplementary Figure 1. ML analysis of angiosperm PEBP genes. Summary tree (upper left), the expanded clade in the figure is indicated in red. Yellow stars indicate large-scale duplication events, while red stars represent intra-specific duplications. The numbers on each node indicate the UFBS values. Collapsed clades correspond to *FT-like* (Figures 1-3) and *BFT/TFL1-like* (Figure 4). The colors of the taxa correspond to the conventions on the left. The scale of the tree is 0.2.

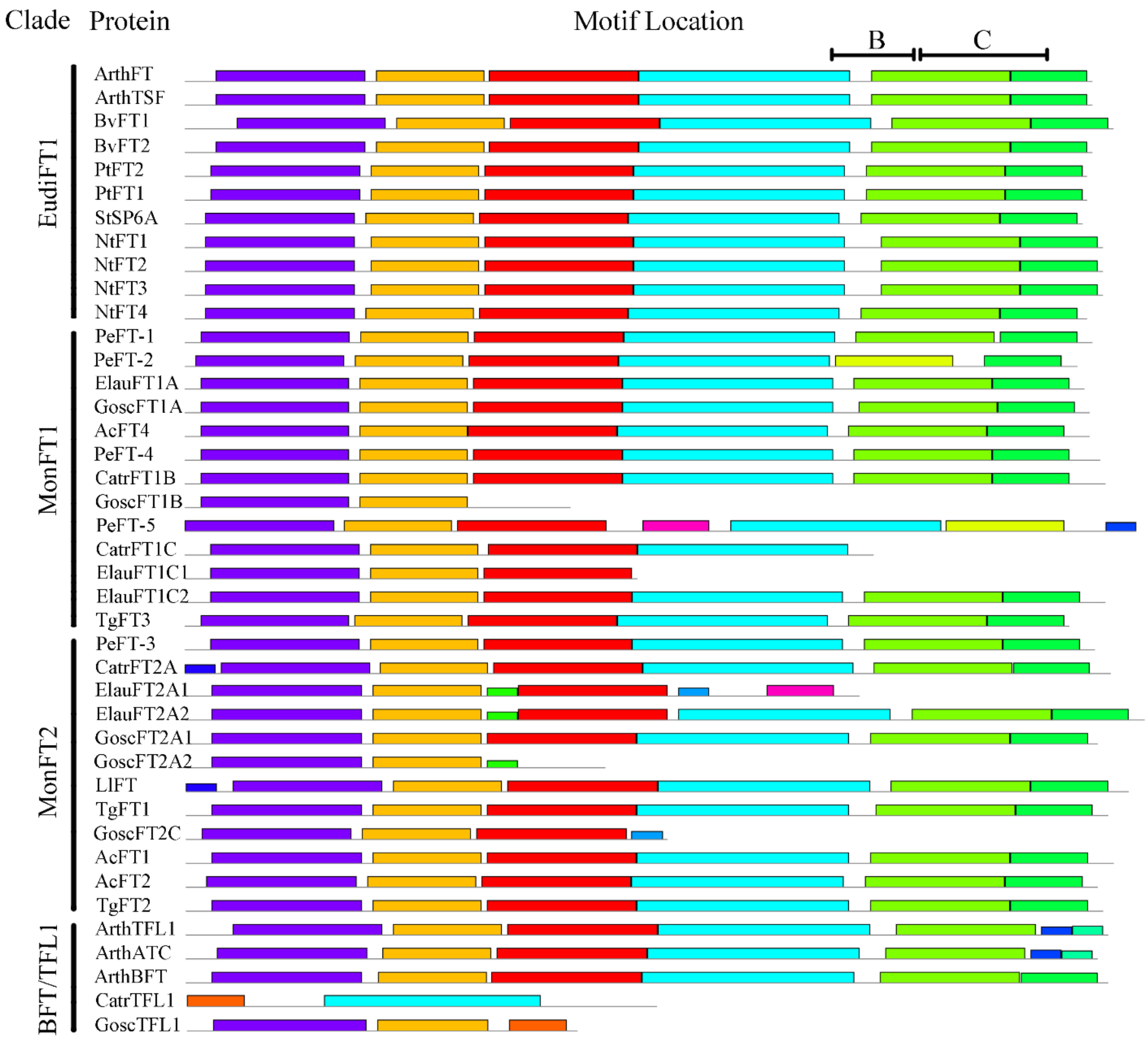

Motif		Motif	
1)	DPLVVGRVIGDVLDPFTRSVSLRVVYNNR	8)	DLRTFY
2)	TNGCELKPSAVVNQPRVEIGG	9)	TAARKR
3)	DLRTFYTLVMVDPDAPSPSNPTLREYLHW	10)	CKVQGGKKLYKFI
4)	LVTDIPATTDASFGNEIVCYESPRPSLGIHRFVFVLFRQLG	11)	FNCQRE
5)	YAPGWRQNFNTRDFAELYNLGSPVAAV	12)	LVTDIP
6)	YFNCQRESGSGGRRF	13)	MEMRRQ
7)	NTVFEPGWRQNFNTRDFAQEYNL	14)	SFFTLVMTDPD

Supplementary Figure 2. MEME analysis of protein motifs. Names to the left indicate the clade to which sequences belong according to Figures 1-4. The upper bars point to the segment B and C reported in *Arabidopsis thaliana* (Anh et al., 2006). The number assigned to each motif and its respective logo are shown in the lower part of the figure. In motif three, the position homologous to Tyr-85 and His-88 of FT and TFL1 of *A. thaliana* respectively is indicated with a red triangle (Hanzawa et al. 2005).

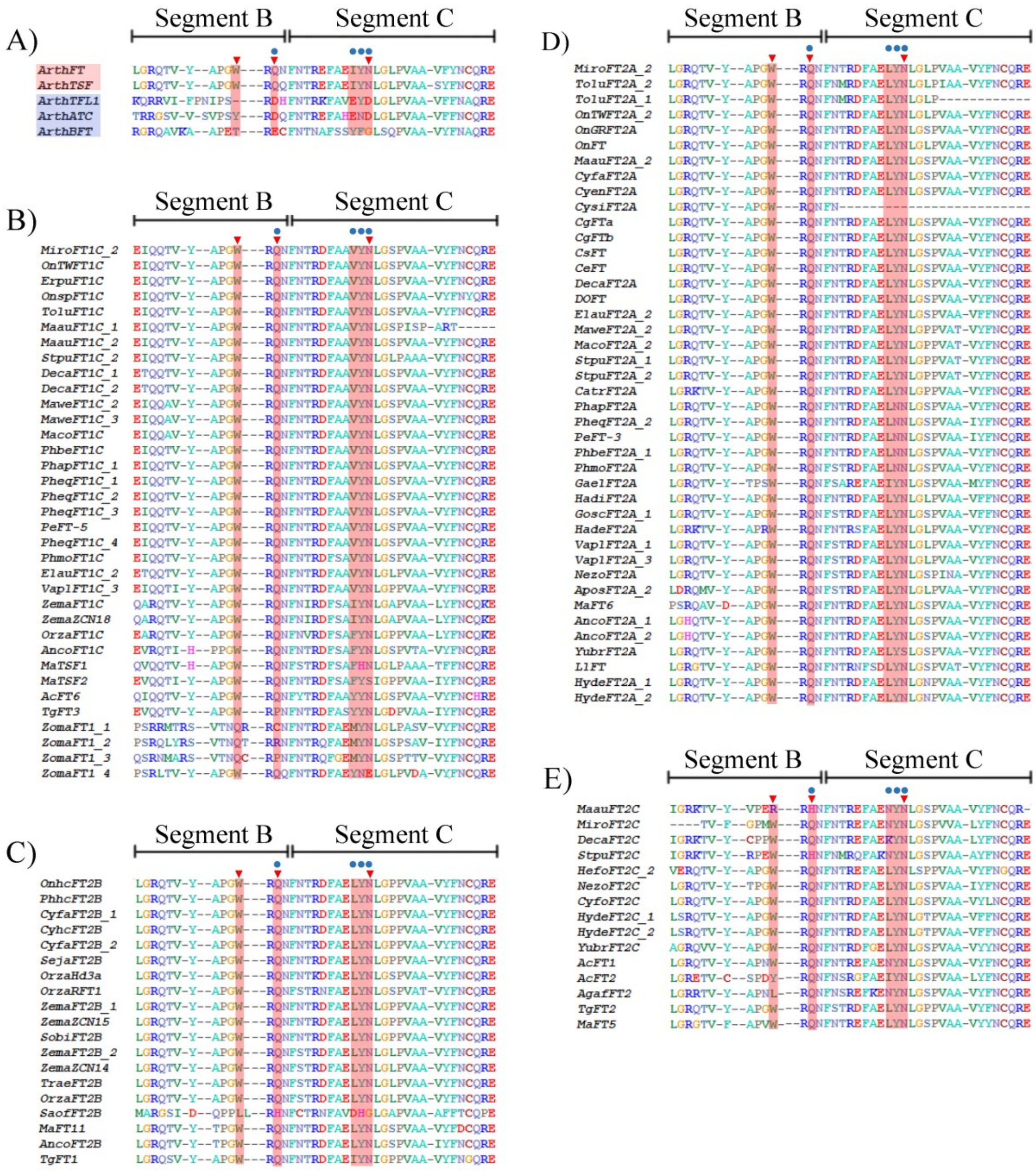
A)
Segment B
Segment C
ArthFT
ArthTSF
ArthTFL1
ArthATC
ArthBFT
B)
Segment B
Segment C
MiroFT1C_2
OnTWFT1C
ErpuFT1C
OnspFT1C
ToluFT1C
MaauFT1C_1
MaauFT1C_2
StpuFT1C_2
DecaFT1C_1
DecaFT1C_2
MaweFT1C_2
MaweFT1C_3
MacoFT1C
PhbeFT1C
PhapFT1C_1
PheqFT1C_1
PheqFT1C_2
PheqFT1C_3
PeFT-5
PheqFT1C_4
PhmoFT1C
ElauFT1C_2
VaplFT1C_3
ZemaFT1C
ZemaZCN18
OrzaFT1C
AncoFT1C
MaTSF1
MaTSF2
AcFT6
TgFT3
ZomaFT1_1
ZomaFT1_2
ZomaFT1_3
ZomaFT1_4
C)
Segment B
Segment C
OnhcFT2B
PhhcFT2B
CyfaFT2B_1
CyhcFT2B
CyfaFT2B_2
SejaFT2B
OrzaHd3a
OrzaRFT1
ZemaFT2B_1
ZemaZCN15
SobiFT2B
ZemaFT2B_2
ZemaZCN14
TraeFT2B
OrzaFT2B
SaofFT2B
MaFT11
AncoFT2B
TgFT1
D)
Segment B
Segment C
MiroFT2A_2
ToluFT2A_2
ToluFT2A_1
OnTWFT2A_2
OnGRFT2A
OnFT
MaauFT2A_2
CyfaFT2A
CyenFT2A
CysiFT2A
CgFTa
CgFTb
CsFT
CeFT
DecaFT2A
DOFT
ElauFT2A_2
MaweFT2A_2
MacoFT2A_2
StpuFT2A_1
StpuFT2A_2
CatrFT2A
PhapFT2A
PheqFT2A_2
PeFT-3
PhbeFT2A_1
PhmoFT2A
GaelFT2A
HadiFT2A
GoscFT2A_1
HadeFT2A
VaplFT2A_1
VaplFT2A_3
NezoFT2A
AposFT2A_2
MaFT6
AncoFT2A_1
AncoFT2A_2
YubrFT2A
LlFT
HydeFT2A_1
HydeFT2A_2
E)
Segment B
Segment C
MaauFT2C
MiroFT2C
DecaFT2C
StpuFT2C
HefoFT2C_2
NezoFT2C
CyfoFT2C
HydeFT2C_1
HydeFT2C_2
YubrFT2C
AcFT1
AcFT2
AgafFT2
TgFT2
MaFT5

Supplementary figure 3. Alignment of fragments B and C of FT-like proteins isolated from monocots. (**A**) PEBP proteins from *Arabidopsis thaliana*, with the exception of MFT. Blue dots (following Ahn et al. 2006) and red triangles (following critical residues identified by Ho and Weigel, 2014) indicate key amino acids distinguishing FT and TFL-like homologues. (**B**) *MonFT1C* clade. (**C**) *MonFT2B* clade. (**D**) *MonFT2A* clade. (**E**) *MonFT2C* clade. In (**A**), the red boxes on the names of the sequences indicate proteins with flowering promoter function, while the blue boxes indicate proteins with repress function.

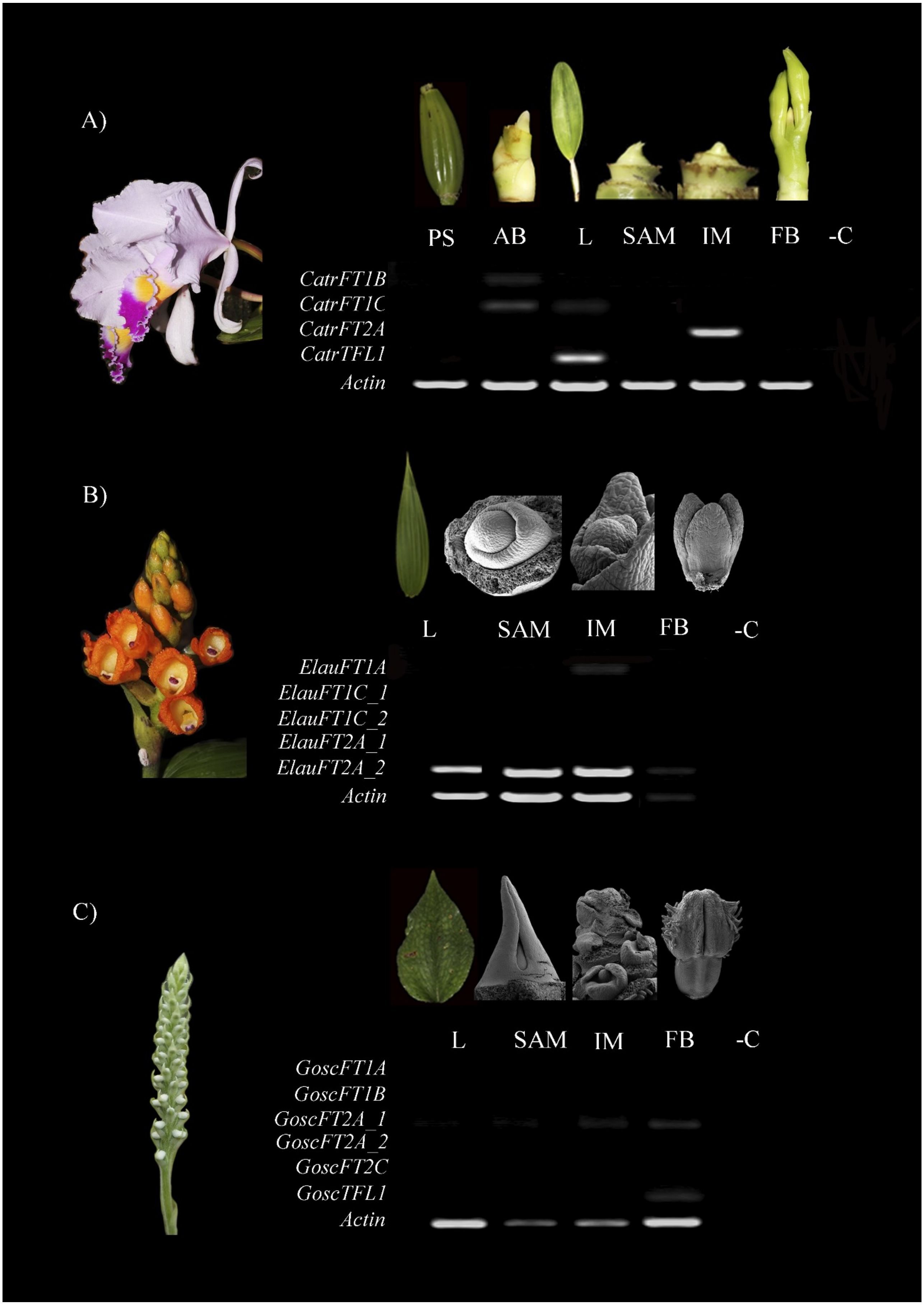
A)
PS
AB
L
SAM
IM
FB
-C
CatrFT1B
CatrFT1C
CatrFT2A
CatrTFL1
Actin
B)
L
SAM
IM
FB
-C
ElauFT1A
ElauFT1C_1
ElauFT1C_2
ElauFT2A_1
ElauFT2A_2
Actin
C)
L
SAM
IM
FB
-C
GoscFT1A
GoscFT1B
GoscFT2A_1
GoscFT2A_2
GoscFT2C
GoscTFL1
Actin

Supplementary Figure 4. Expression analysis of *FT/TFL1-like* genes in selected orchids. Amplifications performed in 30 cycles. (**A**) *Cattleya trianae*. (**B**) *Elleanthus aurantiacus*. (**C**) *Gomphichis scaposa*. AB: Axillary bud, BF: Floral bud, IM: Inflorescence meristem, L: Leaf, PB: Pseudobulb and SAM: Apical vegetative meristem. Each dissection correspond with a light stereoscope photograph. -C indicated the PCR amplification lacking cDNA as a load control.

Supplementary Tables

Supplementary Table 1. Species of selected Neotropical orchids that have new sequenced transcriptome.

**Specie**	**Habit**	**Number of flowers per inflorescence**	**Pseudobulb**	**Voucher code**
Elleanthus aurantiacus	Terrestrial	More than 10	Absent	NP394
Gomphichis scaposa	Terrestrial	More than 30	Absent	NP398
Masdevallia coccinea	Terrestrial	1	Absent	YMB30
Masdevallia wendlandiana	Epiphyte	1	Absent	YMB31
Maxillaria aurea	Terrestrial	Ca. 5	Absent	YMB36
Miltoniopsis roezlii	Epiphyte	Ca. 5	Present	YMB33
Oncidium Gower Ramsey	Epiphyte	More than 20	Present	NP396
Oncidium Twinkle	Epiphyte	More than 20	Present	YMB32
Stelis pusilla	Epiphyte	More than 20	Absent	YMB37<br>NP423
Tolumnia Cherry red *x* Ralph yagh	Epiphyte	Ca. 10	Present	NP397

Supplementary table 2. Primers used for *FT/TFL1-like* gene expression analyses. *Fwd* indicate forward primer. *Rev* indicate reverse primer.

**Specie name**	**Primer name**	**Sequence**	**Tm (°C)**	**Amplicon size (bp)**
Cattleya trianae	*CatrFT1BFwd*	CGTCCATGAACGTGATCTAT	56.4	379
Cattleya trianae	*CatrFT1BRev*	GCAGTTGAAGTACATGGCA	55.2	
Cattleya trianae	*CatrFT1CFwd*	GTAGGTGATGTGCTGGAC	56.1	283
Cattleya trianae	*CatrFT1CRev*	CGATGAATTCCAGCTTGT	51.6	
Cattleya trianae	*CatrFT2AFwd*	GAGTTATAGGTGATGTACTT	52.3	293
Cattleya trianae	*CatrFT2ARev*	CGAAGATGAAGCGGTGTAT	55.2	
Cattleya trianae	*CatrTFL1Fwd*	CACTGGTCATGACTGATC	53.9	169
Cattleya trianae	*CatrTFL1Rev*	CACCGATAGCCTTCGCTT	56.1	
Elleanthus aurantiacus	*ElauFT1AFwd*	GATCATTTAGTGAGGTTA	47.0	289
Elleanthus aurantiacus	*ElauFT1ARev*	GAACTGTCGTAAGAGTGC	53.9	
Elleanthus aurantiacus	*ElauFT1C1/FT1C2Fwd*	GGTGACGTGCTGGATCCT	58.4	270
Elleanthus aurantiacus	*ElauFT1C1Rev*	GCTTAAAGCTTACCAATG	49.3	
Elleanthus aurantiacus	*ElauFT1C2Rev*	GATGAATTCCAGCTAGTG	51.6	276

Elleanthus aurantiacus	*ElauFT2A1/FT2A2Fwd*	GTGATAGGTGATGTACTTGAT	55.5	270
Elleanthus aurantiacus	*ElauFT2A1Rev*	CTTTATACATGTACTCAC	47.0	
Elleanthus aurantiacus	*ElauFT2A2Rev*	GATATAGCGGTGTATGCCTA	56.4	309
Gomphichis scaposa	*GoscFT1AFwd*	GATCATACAGTGAGGTTTAG	54.3	316
Gomphichis scaposa	*GoscFT1ARev*	CGCACTTCTGGCTCAATGA	57.3	
Gomphichis scaposa	*GoscFT1BFwd*	GTCGTCGGCAATGTTGTAG	57.3	147
Gomphichis scaposa	*GoscFT1BRev*	CCACTTACTAGCGTATAA	49.3	
Gomphichis scaposa	*GoscFT2A1/FT2A2Fwd*	GAGTGATAGGCGATGTTCTC	58.4	280
Gomphichis scaposa	*GoscFT2A1Rev*	CGATGTATACCAAGACTC	51.6	
Gomphichis scaposa	*GoscFT2A2Rev*	CTGTGACTAACCAGAGTA	51.6	139
Gomphichis scaposa	*GoscFT2CFwd*	GAGTGATTGGTGACGTGT	53.9	193
Gomphichis scaposa	*GoscFT2CRev*	CTGCAGTGAAGATATTCTCT	54.3	
Gomphichis scaposa	*GoscTFL1Fwd*	GCGAAGTGCTCGAATCCT	56.1	140
Gomphichis scaposa	*GoscTFL1Rev*	CGTCTGGATCGGTCATTA	53.9	

Cattleya trianae	qCatrTFL1Rev	TGCGAAGCCACGTGTATTAAA	54	< 150
Cattleya trianae	qCatrTFL1Fwd	AGCTGGTTCCCTATGAGAGC	54	
Cattleya trianae	qCatrFT2ARev	GGGCTCTCGTAGCACACTAT	54	< 150
Cattleya trianae	qCatrFT2AFwd	AGCCGAGAGTTGAAGTTGGA	54	
Cattleya trianae	qCatrFT1CRev	TGCGGTCCTTCGTAGTAGAC	54	< 150
Cattleya trianae	qCatrFT1CFwd	GGTTGAGATTGGTGGTGGTG	54	
Cattleya trianae	qCatrFT1BRev	TTCCCTCTGGCAGTTGAAGT	54	< 150
Cattleya trianae	qCatrFT1BFwd	AGCCGGGATCCATCGAATAG	54	
Cattleya trianae	qOrchActinFwd	TGCTGGATTCTGGTGATGGT	54	< 150
Cattleya trianae	qOrchActinRev	CAATTTCACGCTCTGCGGTA	54	
Cattleya trianae	18SFwd	GAGAAATCAAAGTTTTTGGGTTCCG	54	< 150
Cattleya trianae	18SRev	CCGTGTTGAGTCAAATTAAGCCGC	54	

Supplementary table 3. List of sequences used in this study.

Gene code	Locus name	Data base	Specie	Family	Order
AcFT1	KC485348.1	NCBI	*Allium cepa*	Amaryllidaceae	Asparagales
AcFT2	KC485349.1	NCBI	*Allium cepa*	Amaryllidaceae	Asparagales
AcFT3	KC485350.1	NCBI	*Allium cepa*	Amaryllidaceae	Asparagales
AcFT4	KC485351.1	NCBI	*Allium cepa*	Amaryllidaceae	Asparagales
AcFT5	KC485352.1	NCBI	*Allium cepa*	Amaryllidaceae	Asparagales
AcFT6	KC485353.1	NCBI	*Allium cepa*	Amaryllidaceae	Asparagales
AgafFT2	PRFO_scaffold_2024105	1KP	*Agapanthus africanus*	Amaryllidaceae	Asparagales
AgteFT1A	KXSK_scaffold_2021615	1KP	*Agave tequila*	Asparagaceae	Asparagales
AmtrFT1	XM_006858577.3	NCBI	*Amborella trichopoda*	Amborellaceae	Amborellales
AmtrFT2	URDJ_scaffold_2034066	1KP	*Amborella trichopoda*	Amborellaceae	Amborellales
AmtrFT3	evm_27.model.AmTr_v1.0_scaffold00066.24	Phytozome	*Amborella tricopoda*	Amborellaceae	Amborellales
AmtrMFT1	evm_27.model.AmTr_v1.0_scaffold00024.325	Phytozome	*Amborella tricopoda*	Amborellaceae	Amborellales
AmtrMFT2	evm_27.model.AmTr_v1.0_scaffold00003.462	Phytozome	*Amborella tricopoda*	Amborellaceae	Amborellales
AmtrTFL1	evm_27.model.AmTr_v1.0_scaffold00114.23	Phytozome	*Amborella tricopoda*	Amborellaceae	Amborellales
AnarBFT	TQKZ_scaffold_2057863	1KP	*Angelica archangelica*	Apiaceae	Apiales
AncoFT1B1	Aco003470.1	Phytozome	*Ananas comosus*	Bromeliaceae	Poales
AncoFT1B2	Aco008070.1	Phytozome	*Ananas comosus*	Bromeliaceae	Poales

AncoFT1C	Aco004692.1	Phytozome	*Ananas comosus*	Bromeliaceae	Poales
AncoFT2A1	HQ343233	NCBI	*Ananas comosus*	Bromeliaceae	Poales
AncoFT2A2	Aco010684.1	Phytozome	*Ananas comosus*	Bromeliaceae	Poales
AncoFT2B	Aco010683.1	Phytozome	*Ananas comosus*	Bromeliaceae	Poales
AncoTFL1_1	Aco031443.1	Phytozome	*Ananas comosus*	Bromeliaceae	Poales
AncoTFL1_2	Aco016718.1	Phytozome	*Ananas comosus*	Bromeliaceae	Poales
ApostBFT	AUTC016670	ORCHIDSTRA	*Apostasia wallichii*	Orchidaceae	Asparagales
ApostFT2A1	AUTC002880	ORCHIDSTRA	*Apostasia wallichii*	Orchidaceae	Asparagales
ApostFT2A2	AUTC002879	ORCHIDSTRA	*Apostasia wallichii*	Orchidaceae	Asparagales
ApshFT1B	Unigene16175_Ap_fb	Orchidbase	*Apostasia shenzhenica*	Orchidaceae	Asparagales
AqcoCEN1	Aqcoe2G140500.1	Phytozome	*Aquilegia coerulea*	Ranunculaceae	Ranunculales
AqcoCEN2	Aqcoe1G447900.1	Phytozome	*Aquilegia coerulea*	Ranunculaceae	Ranunculales
AqcoCEN3	Aqcoe1G447700.1	Phytozome	*Aquilegia coerulea*	Ranunculaceae	Ranunculales
AqcoCEN4	Aqcoe1G447800.1	Phytozome	*Aquilegia coerulea*	Ranunculaceae	Ranunculales
AqcoFT1_1	Aqcoe4G257600.1	Phytozome	*Aquilegia coerulea*	Ranunculaceae	Ranunculales
AqcoFT1_2	Aqcoe4G263600.1	Phytozome	*Aquilegia coerulea*	Ranunculaceae	Ranunculales

AqcoFT1_3	Aqcoe2G432300.1	Phytozome	*Aquilegia coerulea*	Ranunculaceae	Ranunculales
AqcoMFT	Aqcoe3G016200.1	Phytozome	*Aquilegia coerulea*	Ranunculaceae	Ranunculales
ArfiFT1	TRINITY_DN13952_c0_g1_i1	UdeA	*Aristolochia fimbriata*	Aristolochiaceae	Piperales
ArfiMFT1	c12632_g1_i1	UdeA	*Aristolochia fimbriata*	Aristolochiaceae	Piperales
ArfiMFT2	c729_g2_i1	UdeA	*Aristolochia fimbriata*	Aristolochiaceae	Piperales
ArfiMFT3	c729_g1_i1	UdeA	*Aristolochia fimbriata*	Aristolochiaceae	Piperales
ArfiMFT4	TRINITY_DN9162_c4_g6_i2	UdeA	*Aristolochia fimbriata*	Aristolochiaceae	Piperales
ArfiMFT5	TRINITY_DN9601_c6_g7_i1	UdeA	*Aristolochia fimbriata*	Aristolochiaceae	Piperales
ArriFT1	TRINITY_DN19698_c0_g1_i1	UdeA	*Aristolochia ringens*	Aristolochiaceae	Piperales
ArriMFT	TRINITY_DN33419_c0_g1_i1	UdeA	*Aristolochia ringens*	Aristolochiaceae	Piperales
ArthATC	AB024715.1	NCBI	*Arabidopsis thaliana*	Brassicaceae	Brassicales
ArthBFT	NM_125597.2	NCBI	*Arabidopsis thaliana*	Brassicaceae	Brassicales
ArthFT	AB027504.1	NCBI	*Arabidopsis thaliana*	Brassicaceae	Brassicales
ArthMFT	AF147721.1	NCBI	*Arabidopsis thaliana*	Brassicaceae	Brassicales
ArthTFL1	U77674.1	NCBI	*Arabidopsis thaliana*	Brassicaceae	Brassicales
ArthTSF	AB027506.1	NCBI	*Arabidopsis thaliana*	Brassicaceae	Brassicales
AuscMFT	FZJL_scaffold_2000368	1KP	*Austrobaileya scandens*	Austrobaileyaceae	Austrobaileyales

AushMFT	YYPE_scaffold_2007933	1KP	*Austrobaileya chilensis*	Austrobaileyaceae	Austrobaileyales
BofrFT1	comp56561_c0_seq1	UdeA	*Bocconia frutescens*	Papaveraceae	Ranunculales
BofrMFT	comp56255_c0_seq1	UdeA	*Bocconia frutescens*	Papaveraceae	Ranunculales
BrauFT1_1	TRINITY_DN50312_c0_g1_i1	UdeA	*Brunfelsia australis*	Solanaceae	Solanales
BrauFT1_2	TRINITY_DN45017_c0_g1_i1	UdeA	*Brunfelsia australis*	Solanaceae	Solanales
BrauFT1_3	TRINITY_DN45017_c0_g2_i1	UdeA	*Brunfelsia australis*	Solanaceae	Solanales
BrauFT1_4	TRINITY_DN46075_c0_g1_i1	UdeA	*Brunfelsia australis*	Solanaceae	Solanales
BrauFT1_5	TRINITY_DN51421_c0_g1_i1	UdeA	*Brunfelsia australis*	Solanaceae	Solanales
BrauFT1_6	TRINITY_DN79559_c0_g3_i1	UdeA	*Brunfelsia australis*	Solanaceae	Solanales
BrauFT1_7	TRINITY_DN79559_c0_g1_i1	UdeA	*Brunfelsia australis*	Solanaceae	Solanales
BrauFT1_8	TRINITY_DN44401_c0_g1_i1	UdeA	*Brunfelsia australis*	Solanaceae	Solanales
BrauFT1_9	TRINITY_DN44401_c0_g1_i2	UdeA	*Brunfelsia australis*	Solanaceae	Solanales
BrauMFT1	TRINITY_DN81540_c0_g1_i1	UdeA	*Brunfelsia australis*	Solanaceae	Solanales
BrauMFT2	TRINITY_DN25212_c0_g1_i1	UdeA	*Brunfelsia australis*	Solanaceae	Solanales
BvBFT1	HM448916.1	NCBI	*Beta vulgaris*	Amaranthaceae	Caryophyllales
BvCEN1	HM448914.1	NCBI	*Beta vulgaris*	Amaranthaceae	Caryophyllales
BvFT1	HM448910.1	NCBI	*Beta vulgaris*	Amaranthaceae	Caryophyllales

BvFT2	HM448912.1	NCBI	*Beta vulgaris*	Amaranthaceae	Caryophyllales
CapaBFT	evm.model.supercontig_107.28	Phytozome	*Carica papaya*	Caricaceae	Brassicales
CapaCEN	evm.model.supercontig_32.19	Phytozome	*Carica papaya*	Caricaceae	Brassicales
CapaFT1	evm.TU.contig_32595.1	Phytozome	*Carica papaya*	Caricaceae	Brassicales
CapaMFT1	evm.model.supercontig_3387.2	Phytozome	*Carica papaya*	Caricaceae	Brassicales
CapaMFT2	evm.model.supercontig_3.421	Phytozome	*Carica papaya*	Caricaceae	Brassicales
CapaTFL1	evm.TU.contig_45053.1	Phytozome	*Carica papaya*	Caricaceae	Brassicales
CatrFT1B	c14428_g1_i1	UdeA	*Cattleya trianae*	Orchidaceae	Asparagales
CatrFT1C	c4094_g1_i1	UdeA	*Cattleya trianae*	Orchidaceae	Asparagales
CatrFT2A	c7156_g1_i1	UdeA	*Cattleya trianae*	Orchidaceae	Asparagales
CatrTFL1	c25489_g1_i1	UdeA	*Cattleya trianae*	Orchidaceae	Asparagales
CeFT	HM803115.1	NCBI	*Cymbidium ensifolium*	Orchidaceae	Asparagales
CgFTa	HM106985.1	NCBI	*Cymbidium goeringii*	Orchidaceae	Asparagales
CgFTb	HM120863.1	NCBI	*Cymbidium goeringii*	Orchidaceae	Asparagales
ChmaCEN1	XMVD_scaffold_2043252	1KP	*Chelidonium majus*	Papaveraceae	Ranunculales
ChmaCEN2	XMVD_scaffold_2010849	1KP	*Chelidonium majus*	Papaveraceae	Ranunculales
CiquCEN	BGZG_scaffold_2061341	1KP	*Cissus quadrangularis*	Vitaceae	Vitales
CiquFT1	BGZG_scaffold_2052450	1KP	*Cissus quadrangularis*	Vitaceae	Vitales

CsFT	HM120862.1	NCBI	*Cymbidium sinense*	Orchidaceae	Asparagales
CusaBFT	Cucsa.250860.1	Phytozome	*Cucumis sativus*	Cucurbitaceae	Cucurbitales
CusaCEN	Cucsa.385120.1	Phytozome	*Cucumis sativus*	Cucurbitaceae	Cucurbitales
CusaFT1	Cucsa.356340.1	Phytozome	*Cucumis sativus*	Cucurbitaceae	Cucurbitales
CusaMFT1	Cucsa.126210.1	Phytozome	*Cucumis sativus*	Cucurbitaceae	Cucurbitales
CusaMFT2	Cucsa.242690.1	Phytozome	*Cucumis sativus*	Cucurbitaceae	Cucurbitales
CusaTFL1_1	Cucsa.241060.1	Phytozome	*Cucumis sativus*	Cucurbitaceae	Cucurbitales
CusaTFL1_2	Cucsa.083330.1	Phytozome	*Cucumis sativus*	Cucurbitaceae	Cucurbitales
CyenFT1A1	CETC001271	ORCHIDSTRA	*Cymbidium ensifolium*	Orchidaceae	Asparagales
CyenFT1A2	CETC001272	ORCHIDSTRA	*Cymbidium ensifolium*	Orchidaceae	Asparagales
CyenFT1B	CETC024003	ORCHIDSTRA	*Cymbidium ensifolium*	Orchidaceae	Asparagales
CyenFT2A	CETC024410	ORCHIDSTRA	*Cymbidium ensifolium*	Orchidaceae	Asparagales
CyfaFT2A	HQ164434.1	NCBI	*Cymbidium faberi*	Orchidaceae	Asparagales
CyfaFT2B1	JQ796076.1	NCBI	*Cymbidium faberi*	Orchidaceae	Asparagales
CyfaFT2B2	KC138734.1	NCBI	*Cymbidium faberi*	Orchidaceae	Asparagales
CyfoFT1B	CFTC011795	ORCHIDSTRA	*Cypripedium formosanum*	Orchidaceae	Asparagales

CyfoFT2C	CFTC014733	ORCHIDSTRA	*Cypripedium formosanum*	Orchidaceae	Asparagales
CyfoMFT	CFTC022341	ORCHIDSTRA	*Cypripedium formosanum*	Orchidaceae	Asparagales
CyhcFT2B	KF669643.1	NCBI	*Cymbidium* hybrid cultivar	Orchidaceae	Asparagales
CysgFT2C	Unigene90895_Ch_fb	Orchidbase	*Cypripedium singchii*	Orchidaceae	Asparagales
CysgMFT	Unigene103006_Ch_fb	Orchidbase	*Cypripedium singchii*	Orchidaceae	Asparagales
CysiFT1A	CSTC003002	ORCHIDSTRA	*Cymbidium sinense*	Orchidaceae	Asparagales
CysiFT2A	Unigene91310_Cym_fb	Orchidbase	*Cymbidium sinense*	Orchidaceae	Asparagales
DacaBFT	DCAR_020239	Phytozome	*Daucus carota*	Apiaceae	Apiales
DacaCEN1	DCAR_012187	Phytozome	*Daucus carota*	Apiaceae	Apiales
DacaCEN2	DCAR_010360	Phytozome	*Daucus carota*	Apiaceae	Apiales
DacaCEN3	DCAR_006159	Phytozome	*Daucus carota*	Apiaceae	Apiales
DacaFT1	DCAR_023904	Phytozome	*Daucus carota*	Apiaceae	Apiales
DecaFT1A1	XM_020847639.1	NCBI	*Dendrobium catenatum*	Orchidaceae	Asparagales
DecaFT1A2	XM_020847692.1	NCBI	*Dendrobium catenatum*	Orchidaceae	Asparagales
DecaFT1A3	XM_020819681.1	NCBI	*Dendrobium catenatum*	Orchidaceae	Asparagales
DecaFT1B1	XM_020817371.1	NCBI	*Dendrobium catenatum*	Orchidaceae	Asparagales
DecaFT1B2	XM_020846691.1	NCBI	*Dendrobium catenatum*	Orchidaceae	Asparagales
DecaFT1C1	XR_002305210.1	NCBI	*Dendrobium catenatum*	Orchidaceae	Asparagales

DecaFT1C2	XM_020846707.1	NCBI	*Dendrobium catenatum*	Orchidaceae	Asparagales
DecaFT2A	XM_020849885.1	NCBI	*Dendrobium catenatum*	Orchidaceae	Asparagales
DecaFT2C	XM_020839137.1	NCBI	*Dendrobium catenatum*	Orchidaceae	Asparagales
DecaMFT	XM_020843384.1	NCBI	*Dendrobium catenatum*	Orchidaceae	Asparagales
DenoFT1A	DNTC002854	ORCHIDSTRA	*Dendrobium nobile*	Orchidaceae	Asparagales
DenoFT2A	DNTC004082	ORCHIDSTRA	*Dendrobium nobile*	Orchidaceae	Asparagales
DenoMFT	DNTC002178	ORCHIDSTRA	*Dendrobium nobile*	Orchidaceae	Asparagales
DOFT	MF063061.1	NCBI	*Dendrobium* hybrid cultivar	Orchidaceae	Asparagales
DrelFT2C	XZME_scaffold_2030044	1KP	*Drakaea elastica*	Orchidaceae	Asparagales
DrwiBFT	WKSU_scaffold_2129761	1KP	*Drimys winteri*	Winteraceae	Canellales
DrwiCEN	WKSU_scaffold_2031388	1KP	*Drimys winteri*	Winteraceae	Canellales
ElauFT1A	TRINITY_DN16742_c0_g1_i2	UdeA	*Elleanthus aurantiacus*	Orchidaceae	Asparagales
ElauFT1C1	TRINITY_DN1688_c0_g1_i2	UdeA	*Elleanthus aurantiacus*	Orchidaceae	Asparagales
ElauFT1C2	TRINITY_DN1688_c0_g1_i1	UdeA	*Elleanthus aurantiacus*	Orchidaceae	Asparagales
ElauFT2A1	TRINITY_DN22498_c3_g1_i2	UdeA	*Elleanthus aurantiacus*	Orchidaceae	Asparagales
ElauFT2A2	TRINITY_DN22498_c3_g1_i1	UdeA	*Elleanthus aurantiacus*	Orchidaceae	Asparagales
ElauMFT	TRINITY_DN22697_c3_g1_i1	UdeA	*Elleanthus aurantiacus*	Orchidaceae	Asparagales

ErpuFT1B	EPTC070766	ORCHIDSTRA	*Erycina pusilla*	Orchidaceae	Asparagales
ErpuFT1C	EPTC022265	ORCHIDSTRA	*Erycina pusilla*	Orchidaceae	Asparagales
FrveBFT	mrna13304.1-v1.0-hybrid	Phytozome	*Fragaria vesca*	Rosaceae	Rosales
FrveCEN	mrna21992.1-v1.0-hybrid	Phytozome	*Fragaria vesca*	Rosaceae	Rosales
FrveFT1_1	mrna28959.1-v1.0-hybrid	Phytozome	*Fragaria vesca*	Rosaceae	Rosales
FrveFT1_2	mrna21535.1-v1.0-hybrid	Phytozome	*Fragaria vesca*	Rosaceae	Rosales
FrveMFT	mrna09405.1-v1.0-hybrid	Phytozome	*Fragaria vesca*	Rosaceae	Rosales
FrveTFL1	mrna30276.1-v1.0-hybrid	Phytozome	*Fragaria vesca*	Rosaceae	Rosales
GaelFT1B1	GETC021275	ORCHIDSTRA	*Gastrodia elata*	Orchidaceae	Asparagales
GaelFT1B2	GETC027491	ORCHIDSTRA	*Gastrodia elata*	Orchidaceae	Asparagales
GaelFT2A	GETC013754	ORCHIDSTRA	*Gastrodia elata*	Orchidaceae	Asparagales
GafaTFL1	Unigene12193_Ga_fb	Orchidbase	*Galeola faberi*	Orchidaceae	Asparagales
GopuFT1B	VTUS_scaffold_2004082	1KP	*Goodyera pubescens*	Orchidaceae	Asparagales
GopuFT2A	VTUS_scaffold_2031753	1KP	*Goodyera pubescens*	Orchidaceae	Asparagales
GoscFT1A	TRINITY_DN13863_c0_g1_i1	UdeA	*Gomphichis scaposa*	Orchidaceae	Asparagales
GoscFT1B	TRINITY_DN23199_c0_g1_i2	UdeA	*Gomphichis scaposa*	Orchidaceae	Asparagales
GoscFT2A1	TRINITY_DN19348_c0_g1_i1	UdeA	*Gomphichis scaposa*	Orchidaceae	Asparagales

GoscFT2A2	TRINITY_DN19348_c0_g1_i2	UdeA	*Gomphichis scaposa*	Orchidaceae	Asparagales
GoscFT2C	TRINITY_DN34518_c0_g1_i1	UdeA	*Gomphichis scaposa*	Orchidaceae	Asparagales
GoscMFT1	TRINITY_DN14314_c0_g1_i1	UdeA	*Gomphichis scaposa*	Orchidaceae	Asparagales
GoscMFT2	TRINITY_DN14314_c0_g1_i2	UdeA	*Gomphichis scaposa*	Orchidaceae	Asparagales
GoscTFL1	TRINITY_DN30644_c0_g1_i1	UdeA	*Gomphichis scaposa*	Orchidaceae	Asparagales
GtFT1	AB605176.1	NCBI	*Gentiana triflora*	Gentianaceae	Gentianales
GtFT2	AB605177.1	NCBI	*Gentiana triflora*	Gentianaceae	Gentianales
GtTFL1	AB605178.1	NCBI	*Gentiana triflora*	Gentianaceae	Gentianales
HadeFT2A	Unigene18573_Ha_fb	Orchidbase	*Habenaria delavayi*	Orchidaceae	Asparagales
HadeTFL1	Unigene15403_Ha_fb	Orchidbase	*Habenaria delavayi*	Orchidaceae	Asparagales
HadiFT1B	LELS_scaffold_2082772	1KP	*Haemaria discolor*	Orchidaceae	Asparagales
HadiFT2A	LELS_scaffold_2087437	1KP	*Haemaria discolor*	Orchidaceae	Asparagales
HefoFT2A	Unigene85702_Hem	Orchidbase	*Hemipilia forrestii*	Orchidaceae	Asparagales
HefoFT2C1	Unigene102058_Hem	Orchidbase	*Hemipilia forrestii*	Orchidaceae	Asparagales
HefoFT2C2	Unigene82148_Hem	Orchidbase	*Hemipilia forrestii*	Orchidaceae	Asparagales
HefoTFL1	Unigene16939_Hem	Orchidbase	*Hemipilia forrestii*	Orchidaceae	Asparagales

HovuMFT1	AL508112	NCBI	*Hordeum vulgare*	Poaceae	Poales
HovuMFT2	BG417566	NCBI	*Hordeum vulgare*	Poaceae	Poales
HovuTFL1	AB447465	NCBI	*Hordeum vulgare*	Poaceae	Poales
HydeFT1B1	c89267_g1_i1	UdeA	*Hypoxis decumbens*	Hypoxidaceae	Asparagales
HydeFT1B2	c88391_g1_i1	UdeA	*Hypoxis decumbens*	Hypoxidaceae	Asparagales
HydeFT1C	c9010_g1_i1	UdeA	*Hypoxis decumbens*	Hypoxidaceae	Asparagales
HydeFT2	c14809_g1_i1	UdeA	*Hypoxis decumbens*	Hypoxidaceae	Asparagales
HydeFT2A1	c23061_g2_i2	UdeA	*Hypoxis decumbens*	Hypoxidaceae	Asparagales
HydeFT2A2	c23061_g2_i1	UdeA	*Hypoxis decumbens*	Hypoxidaceae	Asparagales
HydeFT2C1	c23061_g1_i2	UdeA	*Hypoxis decumbens*	Hypoxidaceae	Asparagales
HydeFT2C2	c23061_g1_i1	UdeA	*Hypoxis decumbens*	Hypoxidaceae	Asparagales
HydeMFT1	c20931_g1_i1	UdeA	*Hypoxis decumbens*	Hypoxidaceae	Asparagales
HydeMFT2	c20456_g1_i1	UdeA	*Hypoxis decumbens*	Hypoxidaceae	Asparagales
HydeMFT3	c20456_g2_i1	UdeA	*Hypoxis decumbens*	Hypoxidaceae	Asparagales
HydeTFL1_1	c23090_g1_i1	UdeA	*Hypoxis decumbens*	Hypoxidaceae	Asparagales
HydeTFL1_2	c102156_g1_i1	UdeA	*Hypoxis decumbens*	Hypoxidaceae	Asparagales
KaheMFT	NWMY_scaffold_2062140	1KP	*Kadsura heteroclita*	Schisandraceae	Austrobaileyales

LlFT	MG121856.1	NCBI	*Lilium longiflorum*	Liliaceae	Liliales
LopeTFL1	AF316419	NCBI	*Lolium perenne*	Poaceae	Poales
MaauFT1A 1	TRINITY_DN14829_c0_g1_i1	UdeA	*Maxillaria aurea*	Orchidaceae	Asparagales
MaauFT1A 2	TRINITY_DN14829_c0_g1_i2	UdeA	*Maxillaria aurea*	Orchidaceae	Asparagales
MaauFT1B4	TRINITY_DN16463_c0_g3_i2	UdeA	*Maxillaria aurea*	Orchidaceae	Asparagales
MaauFT1B1	TRINITY_DN15080_c0_g1_i3	UdeA	*Maxillaria aurea*	Orchidaceae	Asparagales
MaauFT1B2	TRINITY_DN15080_c0_g1_i1	UdeA	*Maxillaria aurea*	Orchidaceae	Asparagales
MaauFT1B3	TRINITY_DN15080_c0_g1_i2	UdeA	*Maxillaria aurea*	Orchidaceae	Asparagales
MaauFT1C1	TRINITY_DN15069_c0_g1_i1	UdeA	*Maxillaria aurea*	Orchidaceae	Asparagales
MaauFT1C2	TRINITY_DN15069_c0_g1_i2	UdeA	*Maxillaria aurea*	Orchidaceae	Asparagales
MaauFT2A 1	TRINITY_DN15519_c0_g1_i2	UdeA	*Maxillaria aurea*	Orchidaceae	Asparagales
MaauFT2A 2	TRINITY_DN15519_c0_g1_i1	UdeA	*Maxillaria aurea*	Orchidaceae	Asparagales
MaauFT1A 3	TRINITY_DN14829_c0_g1_i3	UdeA	*Maxillaria aurea*	Orchidaceae	Asparagales
MaauFT2C	TRINITY_DN25818_c0_g1_i1	UdeA	*Maxillaria aurea*	Orchidaceae	Asparagales
MacoFT1A	TRINITY_DN53367_c0_g1_i1	UdeA	*Masdevallia coccinea*	Orchidaceae	Asparagales
MacoFT1B	TRINITY_DN36146_c0_g1_i1	UdeA	*Masdevallia coccinea*	Orchidaceae	Asparagales

MacoFT1C	TRINITY_DN23174_c0_g1_i2	UdeA	*Masdevallia coccinea*	Orchidaceae	Asparagales
MacoFT2A 1	TRINITY_DN23542_c0_g1_i3	UdeA	*Masdevallia coccinea*	Orchidaceae	Asparagales
MacoFT2A 2	TRINITY_DN23542_c0_g1_i2	UdeA	*Masdevallia coccinea*	Orchidaceae	Asparagales
MaFT1	GSMUA_Achr9T07900_001	Phytozome	*Musa acuminata*	Musaceae	Zingiberales
MaFT10	GSMUA_Achr2T20900_001	Phytozome	*Musa acuminata*	Musaceae	Zingiberales
MaFT11	GSMUA_Achr10T21760_001	Phytozome	*Musa acuminata*	Musaceae	Zingiberales
MaFT12	GSMUA_Achr5T06420_001	Phytozome	*Musa acuminata*	Musaceae	Zingiberales
MaFT3	GSMUA_Achr5T06600_001	Phytozome	*Musa acuminata*	Musaceae	Zingiberales
MaFT4	GSMUA_Achr2T19360_001	Phytozome	*Musa acuminata*	Musaceae	Zingiberales
MaFT5	GSMUA_Achr4T08540_001	Phytozome	*Musa acuminata*	Musaceae	Zingiberales
MaFT6	GSMUA_Achr2T19310_001	Phytozome	*Musa acuminata*	Musaceae	Zingiberales
MaFT7	GSMUA_Achr2T05870_001	Phytozome	*Musa acuminata*	Musaceae	Zingiberales
MaFT9	GSMUA_Achr3T02730_001	Phytozome	*Musa acuminata*	Musaceae	Zingiberales
MaTSF1	GSMUA_Achr1T17050_001	Phytozome	*Musa acuminata*	Musaceae	Zingiberales
MaTSF2	GSMUA_Achr3T21510_001	Phytozome	*Musa acuminata*	Musaceae	Zingiberales
MaweFT1A	TRINITY_DN34103_c0_g1_i1	UdeA	*Masdevallia wendlandiana*	Orchidaceae	Asparagales
MaweFT1B	TRINITY_DN4104_c0_g1_i1	UdeA	*Masdevallia wendlandiana*	Orchidaceae	Asparagales

MaweFT1C 1	TRINITY_DN12844_c0_g1_i1	UdeA	*Masdevallia wendlandiana*	Orchidaceae	Asparagales
MaweFT1C 2	TRINITY_DN12844_c0_g1_i2	UdeA	*Masdevallia wendlandiana*	Orchidaceae	Asparagales
MaweFT1C 3	TRINITY_DN12844_c0_g1_i3	UdeA	*Masdevallia wendlandiana*	Orchidaceae	Asparagales
MaweFT2A 1	TRINITY_DN12012_c0_g1_i1	UdeA	*Masdevallia wendlandiana*	Orchidaceae	Asparagales
MaweFT2A 2	TRINITY_DN12012_c0_g1_i3	UdeA	*Masdevallia wendlandiana*	Orchidaceae	Asparagales
MaweFT2A 3	TRINITY_DN12012_c0_g1_i2	UdeA	*Masdevallia wendlandiana*	Orchidaceae	Asparagales
MaweTFL1	TRINITY_DN6371_c0_g1_i1	UdeA	*Masdevallia wendlandiana*	Orchidaceae	Asparagales
MayuFT1A 1	JSAG_scaffold_2045808	1KP	*Masdevallia yuangensis*	Orchidaceae	Asparagales
MayuFT1A 2	JSAG_scaffold_2073319	1KP	*Masdevallia yuangensis*	Orchidaceae	Asparagales
MayuFT1B	JSAG_scaffold_2002272	1KP	*Masdevallia yuangensis*	Orchidaceae	Asparagales
MayuFT2A 1	JSAG_scaffold_2074961	1KP	*Masdevallia yuangensis*	Orchidaceae	Asparagales
MayuFT2A 2	JSAG_scaffold_2065793	1KP	*Masdevallia yuangensis*	Orchidaceae	Asparagales
MetrBFT	Medtr0020s0120.1	Phytozome	*Medicago truncatula*	Fabaceae	Fabales
MetrCEN	Medtr2g086270.1	Phytozome	*Medicago truncatula*	Fabaceae	Fabales
MetrFT1_1	Medtr7g085040.1	Phytozome	*Medicago truncatula*	Fabaceae	Fabales
MetrFT1_2	Medtr7g025450.1	Phytozome	*Medicago truncatula*	Fabaceae	Fabales

MetrFT1_3	Medtr7g006630.1	Phytozome	*Medicago truncatula*	Fabaceae	Fabales
MetrFT1_4	Medtr7g006690.1	Phytozome	*Medicago truncatula*	Fabaceae	Fabales
MetrFT1_5	Medtr7g085020.1	Phytozome	*Medicago truncatula*	Fabaceae	Fabales
MetrFT1_6	Medtr7g084970.1	Phytozome	*Medicago truncatula*	Fabaceae	Fabales
MetrFT1_7	Medtr6g033040.1	Phytozome	*Medicago truncatula*	Fabaceae	Fabales
MetrMFT	Medtr8g106840.1	Phytozome	*Medicago truncatula*	Fabaceae	Fabales
MetrTFL1_1	Medtr7g104460.1	Phytozome	*Medicago truncatula*	Fabaceae	Fabales
MetrTFL1_2	Medtr1g060190.1	Phytozome	*Medicago truncatula*	Fabaceae	Fabales
MiroFT1A1	TRINITY_DN17560_c0_g1_i1	UdeA	*Miltoniopsis roezlii*	Orchidaceae	Asparagales
MiroFT1A2	TRINITY_DN10084_c0_g1_i1	UdeA	*Miltoniopsis roezlii*	Orchidaceae	Asparagales
MiroFT1B1	TRINITY_DN24380_c0_g1_i1	UdeA	*Miltoniopsis roezlii*	Orchidaceae	Asparagales
MiroFT1B2	TRINITY_DN15222_c0_g1_i3	UdeA	*Miltoniopsis roezlii*	Orchidaceae	Asparagales
MiroFT1C1	TRINITY_DN6529_c0_g1_i1	UdeA	*Miltoniopsis roezlii*	Orchidaceae	Asparagales
MiroFT1C2	TRINITY_DN6529_c0_g1_i2	UdeA	*Miltoniopsis roezlii*	Orchidaceae	Asparagales
MiroFT2A1	TRINITY_DN17260_c4_g1_i2	UdeA	*Miltoniopsis roezlii*	Orchidaceae	Asparagales
MiroFT2A2	TRINITY_DN17260_c4_g1_i1	UdeA	*Miltoniopsis roezlii*	Orchidaceae	Asparagales
MiroFT2A3	TRINITY_DN17260_c4_g1_i4	UdeA	*Miltoniopsis roezlii*	Orchidaceae	Asparagales

MiroFT2C	TRINITY_DN17322_c1_g5_i5	UdeA	*Miltoniopsis roezlii*	Orchidaceae	Asparagales
MiroTFL1	TRINITY_DN28320_c0_g1_i1	UdeA	*Miltoniopsis roezlii*	Orchidaceae	Asparagales
MuacBFT1	GSMUA_Achr5T20040_001	Phytozome	*Musa acuminata*	Musaceae	Zingiberales
MuacBFT2	GSMUA_Achr11T21720_001	Phytozome	*Musa acuminata*	Musaceae	Zingiberales
MuacMFT1	GSMUA_Achr6T09410_001	Phytozome	*Musa acuminata*	Musaceae	Zingiberales
MuacMFT2	GSMUA_AchrUn_randomT20510_001	Phytozome	*Musa acuminata*	Musaceae	Zingiberales
MuacMFT3	GSMUA_Achr3T20880_001	Phytozome	*Musa acuminata*	Musaceae	Zingiberales
MuacTFL1_1	GSMUA_Achr10T15580_001	Phytozome	*Musa acuminata*	Musaceae	Zingiberales
MuacTFL1_2	GSMUA_Achr3T03070_001	Phytozome	*Musa acuminata*	Musaceae	Zingiberales
MuacTFL1_3	GSMUA_Achr10T10290_001	Phytozome	*Musa acuminata*	Musaceae	Zingiberales
MuacTFL1_4	GSMUA_Achr3T22720_001	Phytozome	*Musa acuminata*	Musaceae	Zingiberales
MuacTFL1_5	GSMUA_Achr8T07730_001	Phytozome	*Musa acuminata*	Musaceae	Zingiberales
MyfrBFT1	OBPL_scaffold_2032672	1KP	*Myristica fragrans*	Myristicaceae	Magnoliales
MyfrBFT2	OBPL_scaffold_2005500	1KP	*Myristica fragrans*	Myristicaceae	Magnoliales
NemaFT1A	Unigene3549_Ne_fb	Orchidbase	*Neuwiedia malipoensis*	Orchidaceae	Asparagales
NemaFT2C	Unigene126803_Ne_fb	Orchidbase	*Neuwiedia malipoensis*	Orchidaceae	Asparagales

NemaMFT	Unigene133751_Ne_fb	Orchidbase	*Neuwiedia malipoensis*	Orchidaceae	Asparagales
NezoFT1A	NZTC028978	ORCHIDSTRA	*Neuwiedia zollingeri*	Orchidaceae	Asparagales
NezoFT1B	NZTC007962	ORCHIDSTRA	*Neuwiedia zollingeri*	Orchidaceae	Asparagales
NezoFT1C	NZTC020018	ORCHIDSTRA	*Neuwiedia zollingeri*	Orchidaceae	Asparagales
NezoFT2A	NZTC015683	ORCHIDSTRA	*Neuwiedia zollingeri*	Orchidaceae	Asparagales
NezoFT2C	NZTC028903	ORCHIDSTRA	*Neuwiedia zollingeri*	Orchidaceae	Asparagales
NezoMFT	NZTC024620	ORCHIDSTRA	*Neuwiedia zollingeri*	Orchidaceae	Asparagales
NitaBFT	AF145259	NCBI	*Nicotiana tabacum*	Solanaceae	Solanales
NitaCEN1	AF145260	NCBI	*Nicotiana tabacum*	Solanaceae	Solanales
NitaCEN2	AF145261	NCBI	*Nicotiana tabacum*	Solanaceae	Solanales
NitaCEN3	AF145262	NCBI	*Nicotiana tabacum*	Solanaceae	Solanales
NtFT1	JX679067	NCBI	*Nicotiana tabacum*	Solanaceae	Solanales
NtFT2	JX679068	NCBI	*Nicotiana tabacum*	Solanaceae	Solanales
NtFT3	JX679069	NCBI	*Nicotiana tabacum*	Solanaceae	Solanales
NtFT4	JX679070	NCBI	*Nicotiana tabacum*	Solanaceae	Solanales
OnciFT1A	TRINITY_DN22572_c0_g1_i1	UdeA	*Oncidium* Gower Ramsey	Orchidaceae	Asparagales

OnciFT2A	TRINITY_DN27370_c0_g1_i1	UdeA	*Oncidium* Gower Ramsey	Orchidaceae	Asparagales
OnFT	KJ909968.1	NCBI	*Oncidium* Gower Ramsey	Orchidaceae	Asparagales
OnGRFT2A	EU583502.1	NCBI	*Oncidium* Gower Ramsey	Orchidaceae	Asparagales
OnhcFT2B	KF669642.1	NCBI	*Oncidium* hybrid cultivar	Orchidaceae	Asparagales
OnsPFT1C	CNTZ_scaffold_2040115	1KP	*Oncidium sphacelatum*	Orchidaceae	Asparagales
OnTFL1	KM233713.1	NCBI	*Oncidium* Gower Ramsey	Orchidaceae	Asparagales
OnTWFT1 A1	TRINITY_DN28096_c0_g1_i1	UdeA	*Oncidium* Twinkle	Orchidaceae	Asparagales
OnTWFT1 A2	TRINITY_DN13213_c0_g1_i1	UdeA	*Oncidium* Twinkle	Orchidaceae	Asparagales
OnTWFT1B	TRINITY_DN29823_c0_g1_i1	UdeA	*Oncidium* Twinkle	Orchidaceae	Asparagales
OnTWFT1C	TRINITY_DN15941_c0_g1_i1	UdeA	*Oncidium* Twinkle	Orchidaceae	Asparagales
OnTWFT2 A1	TRINITY_DN14463_c0_g1_i2	UdeA	*Oncidium* Twinkle	Orchidaceae	Asparagales
OnTWFT2 A2	TRINITY_DN14463_c0_g1_i1	UdeA	*Oncidium* Twinkle	Orchidaceae	Asparagales
OnTWTFL1	TRINITY_DN4222_c0_g1_i1	UdeA	*Oncidium* Twinkle	Orchidaceae	Asparagales
OpspFT1B1	OSTC004790	ORCHIDSTR A	*Ophrys sphegodes*	Orchidaceae	Asparagales
OpspFT1B2	OSTC004791	ORCHIDSTR A	*Ophrys sphegodes*	Orchidaceae	Asparagales

OrzaFT1A1	XM_015769366.1	NCBI	*Oryza sativa*	Poaceae	Poales
OrzaFT1A2	XM_015786342.1	NCBI	*Oryza sativa*	Poaceae	Poales
OrzaFT1A3	LOC_Os01g54490	Rice Genome Annotation Project	*Oryza sativa*	Poaceae	Poales
OrzaFT1A4	XM_015785027.1	NCBI	*Oryza sativa*	Poaceae	Poales
OrzaFT1B1	XM_015777901.1	NCBI	*Oryza sativa*	Poaceae	Poales
OrzaFT1B2	XM_015771743.1	NCBI	*Oryza sativa*	Poaceae	Poales
OrzaFT1B3	XM_015762083.1	NCBI	*Oryza sativa*	Poaceae	Poales
OrzaFT1C	XM_015756421.1	NCBI	*Oryza sativa*	Poaceae	Poales
OrzaFT2B	XM_015756406.1	NCBI	*Oryza sativa*	Poaceae	Poales
OrzaHd3a	AB052944.1	NCBI	*Oryza sativa*	Poaceae	Poales
OrzaMFT1	XM_015779388.1	NCBI	*Oryza sativa*	Poaceae	Poales
OrzaMFT2	LOC_Os06g30370	Rice Genome Annotation Project	*Oryza sativa*	Poaceae	Poales
OrzaMFT3	AK107056	NCBI	*Oryza sativa*	Poaceae	Poales
OrzaRFT1	LOC_Os06g06300.1	Rice Genome Annotation Project	*Oryza sativa*	Poaceae	Poales
OrzaTFL1_1	XM_015762401.1	NCBI	*Oryza sativa*	Poaceae	Poales
OrzaTFL1_2	XM_015768632.1	NCBI	*Oryza sativa*	Poaceae	Poales
OrzaTFL1_3	XM_015778658.1	NCBI	*Oryza sativa*	Poaceae	Poales
OrzaTFL1_4	AF159883	NCBI	*Oryza sativa*	Poaceae	Poales
OrzaTFL1_5	AF159882	NCBI	*Oryza sativa*	Poaceae	Poales

PaarFT1B	Unigene93784_Pa_fb	Orchidbase	*Paphiopedilum armeniacum*	Orchidaceae	Asparagales
PaarTFL1	Unigene113364_Pa_fb	Orchidbase	*Paphiopedilum armeniacum*	Orchidaceae	Asparagales
PaFT1	KJ609179.1	NCBI	*Phalaenopsis aphrodite*	Orchidaceae	Asparagales
PaseBFT	JSVC_scaffold_2014753	1KP	*Papaver setigerum*	Papaveraceae	Ranunculales
PaseCEN1	JSVC_scaffold_2157342	1KP	*Papaver setigerum*	Papaveraceae	Ranunculales
PaseCEN2	STDO_scaffold_2022850	1KP	*Papaver setigerum*	Papaveraceae	Ranunculales
PaseCEN3	STDO_scaffold_2149466	1KP	*Papaver setigerum*	Papaveraceae	Ranunculales
PaseCEN4	STDO_scaffold_2040888	1KP	*Papaver setigerum*	Papaveraceae	Ranunculales
PaseFT1_1	MLPX_scaffold_2006863	1KP	*Papaver setigerum*	Papaveraceae	Ranunculales
PaseFT1_2	MLPX_scaffold_2006865	1KP	*Papaver setigerum*	Papaveraceae	Ranunculales
PaseFT1_3	MLPX_scaffold_2006864	1KP	*Papaver setigerum*	Papaveraceae	Ranunculales
PaseFT1_4	MLPX_scaffold_2006866	1KP	*Papaver setigerum*	Papaveraceae	Ranunculales
PaseFT1_5	STDO_scaffold_2030816	1KP	*Papaver setigerum*	Papaveraceae	Ranunculales
PaseFT1_6	EPRK_scaffold_2026376	1KP	*Papaver setigerum*	Papaveraceae	Ranunculales
PaseFT1_7	FNXH_scaffold_2025908	1KP	*Papaver setigerum*	Papaveraceae	Ranunculales
PaseFT1_8	STDO_scaffold_2000001	1KP	*Papaver setigerum*	Papaveraceae	Ranunculales

PaseMFT1	FNXH_scaffold_2005900	1KP	*Papaver setigerum*	Papaveraceae	Ranunculales
PaseMFT2	MLPX_scaffold_2006707	1KP	*Papaver setigerum*	Papaveraceae	Ranunculales
PeFT1	PEQU_33459	Orchidbase	*Phalaenopsis equestris*	Orchidaceae	Asparagales
PeFT2	PEQU_08401	Orchidbase	*Phalaenopsis equestris*	Orchidaceae	Asparagales
PeFT3	PEQU_19304	Orchidbase	*Phalaenopsis equestris*	Orchidaceae	Asparagales
PeFT4	PEQU_16731	Orchidbase	*Phalaenopsis equestris*	Orchidaceae	Asparagales
PeFT5	PEQU_15502	Orchidbase	*Phalaenopsis equestris*	Orchidaceae	Asparagales
PeFT6	PEQU_33463	Orchidbase	*Phalaenopsis equestris*	Orchidaceae	Asparagales
PeFT7	PEQU_06787	Orchidbase	*Phalaenopsis equestris*	Orchidaceae	Asparagales
PeMFT	PEQU_06435	Orchidbase	*Phalaenopsis equestris*	Orchidaceae	Asparagales
PhapFT1A	PATC132794	ORCHIDSTRA	*Phalaenopsis aphrodite*	Orchidaceae	Asparagales
PhapFT1B	PATC058994	ORCHIDSTRA	*Phalaenopsis aphrodite*	Orchidaceae	Asparagales
PhapFT1C1	PATC027165	ORCHIDSTRA	*Phalaenopsis aphrodite*	Orchidaceae	Asparagales
PhapFT1C2	PATC046164	ORCHIDSTRA	*Phalaenopsis aphrodite*	Orchidaceae	Asparagales
PhapFT2A	PATC022841	ORCHIDSTRA	*Phalaenopsis aphrodite*	Orchidaceae	Asparagales
PhapMFT	PATC134478	ORCHIDSTRA	*Phalaenopsis aphrodite*	Orchidaceae	Asparagales
PhbeFT1A1	PBTC029555	ORCHIDSTRA	*Phalaenopsis bellina*	Orchidaceae	Asparagales

PhbeFT1A2	PBTC032059	ORCHIDSTRA	*Phalaenopsis bellina*	Orchidaceae	Asparagales
PhbeFT1B	PBTC025055	ORCHIDSTRA	*Phalaenopsis bellina*	Orchidaceae	Asparagales
PhbeFT1C	PBTC045435	ORCHIDSTRA	*Phalaenopsis bellina*	Orchidaceae	Asparagales
PhbeFT2A1	PBTC040134	ORCHIDSTRA	*Phalaenopsis bellina*	Orchidaceae	Asparagales
PhbeFT2A2	PBTC005504	ORCHIDSTRA	*Phalaenopsis bellina*	Orchidaceae	Asparagales
PhbeMFT1	PBTC005650	ORCHIDSTRA	*Phalaenopsis bellina*	Orchidaceae	Asparagales
PhbeMFT2	PBTC022225	ORCHIDSTRA	*Phalaenopsis bellina*	Orchidaceae	Asparagales
PheqFT1A1	PETC031734	ORCHIDSTRA	*Phalaenopsis equestris*	Orchidaceae	Asparagales
PheqFT1A2	XM_020743463.1	NCBI	*Phalaenopsis equestris*	Orchidaceae	Asparagales
PheqFT1B1	XM_020728915.1	NCBI	*Phalaenopsis equestris*	Orchidaceae	Asparagales
PheqFT1B2	PETC012131	ORCHIDSTRA	*Phalaenopsis equestris*	Orchidaceae	Asparagales
PheqFT1B3	XM_020736438.1	NCBI	*Phalaenopsis equestris*	Orchidaceae	Asparagales
PheqFT1C1	XM_020721263.1	NCBI	*Phalaenopsis equestris*	Orchidaceae	Asparagales
PheqFT1C2	XM_020721262.1	NCBI	*Phalaenopsis equestris*	Orchidaceae	Asparagales
PheqFT1C3	XM_020721260.1	NCBI	*Phalaenopsis equestris*	Orchidaceae	Asparagales
PheqFT1C4	XM_020721261.1	NCBI	*Phalaenopsis equestris*	Orchidaceae	Asparagales

PheqFT2A1	PETC020741	ORCHIDSTRA	*Phalaenopsis equestris*	Orchidaceae	Asparagales
PheqFT2A2	XM_020740221.1	NCBI	*Phalaenopsis equestris*	Orchidaceae	Asparagales
PheqMFT	XM_020716332.1	NCBI	*Phalaenopsis equestris*	Orchidaceae	Asparagales
PhFT	JX162558.1	NCBI	*Phalaenopsis* Fortune Saltzman	Orchidaceae	Asparagales
PhhcFT2B	KC138805.1	NCBI	*Phalaenopsis* hybrid cultivar	Orchidaceae	Asparagales
PhluFT1A1	PLTC041567	ORCHIDSTRA	*Phalaenopsis lueddemanniana*	Orchidaceae	Asparagales
PhluFT1A2	PLTC012154	ORCHIDSTRA	*Phalaenopsis lueddemanniana*	Orchidaceae	Asparagales
PhluFT1A3	PLTC040993	ORCHIDSTRA	*Phalaenopsis lueddemanniana*	Orchidaceae	Asparagales
PhluFT1B1	PLTC029443	ORCHIDSTRA	*Phalaenopsis lueddemanniana*	Orchidaceae	Asparagales
PhluFT1B2	PLTC035491	ORCHIDSTRA	*Phalaenopsis lueddemanniana*	Orchidaceae	Asparagales
PhluFT1C	PLTC025259	ORCHIDSTRA	*Phalaenopsis lueddemanniana*	Orchidaceae	Asparagales
PhluFT2A	PLTC045838	ORCHIDSTRA	*Phalaenopsis lueddemanniana*	Orchidaceae	Asparagales
PhluMFT	PLTC005308	ORCHIDSTRA	*Phalaenopsis lueddemanniana*	Orchidaceae	Asparagales
PhmoFT1A1	PMTC000546	ORCHIDSTRA	*Phalaenopsis modesta*	Orchidaceae	Asparagales

PhmoFT1A2	PMTC002321	ORCHIDSTRA	*Phalaenopsis modesta*	Orchidaceae	Asparagales
PhmoFT1C	PMTC004339	ORCHIDSTRA	*Phalaenopsis modesta*	Orchidaceae	Asparagales
PhmoFT2A	PMTC014557	ORCHIDSTRA	*Phalaenopsis modesta*	Orchidaceae	Asparagales
PhscMFT	PSTC047386	ORCHIDSTRA	*Phalaenopsis schilleriana*	Orchidaceae	Asparagales
PimeMFT1	MAGPIE:pme_PMERO1JP_Mira--PMEROJP1_rep_c21879	Phytometasyn	*Piper methysticum*	Piperaceae	Piperales
PimeMFT2	MAGPIE:pme_PMERO1JP_Mira--PMEROJP1_rep_c13102	Phytometasyn	*Piper methysticum*	Piperaceae	Piperales
PimeMFT3	MAGPIE:pme_PMERO1JP_Mira--PMEROJP1_rep_c15674	Phytometasyn	*Piper methysticum*	Piperaceae	Piperales
PlocMFT	MAGPIE:poc_POCLF1PC_Mira--POCLF1PC_c12569	Phytometasyn	*Platanus occidentalis*	Platanaceae	Proteales
PotrBFT	Potri.015G141300.1	Phytozome	*Populus trichocarpa*	Salicaceae	Malpighiales
PotrCEN1	Potri.004G203900.1	Phytozome	*Populus trichocarpa*	Salicaceae	Malpighiales
PotrCEN2	Potri.009G165100.1	Phytozome	*Populus trichocarpa*	Salicaceae	Malpighiales
PotrMFT	Potri.015G041000.1	Phytozome	*Populus trichocarpa*	Salicaceae	Malpighiales
PtFT1	Potri.008G077700.1	Phytozome	*Populus trichocarpa*	Salicaceae	Malpighiales
PtFT2	Potri.010G179700.1	Phytozome	*Populus trichocarpa*	Salicaceae	Malpighiales
PtFT3	Potri.010G179900.1	Phytozome	*Populus trichocarpa*	Salicaceae	Malpighiales
PtFT4	Potri.002G210200.1	Phytozome	*Populus trichocarpa*	Salicaceae	Malpighiales

SaofFT1A	DN195601	NCBI	*Saccharum officinarum*	Poaceae	Poales
SaofTFL1	CF571229	NCBI	*Saccharum officinarum*	Poaceae	Poales
SejaFT2B	KF669644.1	NCBI	*Sedirea japonica*	Orchidaceae	Asparagales
SobiFT1A1	XM_002451782	NCBI	*Sorghum bicolor*	Poaceae	Poales
SobiFT1A2	XM_002446227	NCBI	*Sorghum bicolor*	Poaceae	Poales
SobiFT1A3	XM_002438506	NCBI	*Sorghum bicolor*	Poaceae	Poales
SobiFT1B1	XM_002454089	NCBI	*Sorghum bicolor*	Poaceae	Poales
SobiFT1B2	XM_002443040	NCBI	*Sorghum bicolor*	Poaceae	Poales
SobiFT2B	XM_002436464	NCBI	*Sorghum bicolor*	Poaceae	Poales
SobiFT6	XM_002456309	NCBI	*Sorghum bicolor*	Poaceae	Poales
SobiMFT	XM_002457449	NCBI	*Sorghum bicolor*	Poaceae	Poales
SobiTFL1_1	XM_002442763	NCBI	*Sorghum bicolor*	Poaceae	Poales
SobiTFL1_2	XM_002450238	NCBI	*Sorghum bicolor*	Poaceae	Poales
SobiTFL1_3	XM_002453886	NCBI	*Sorghum bicolor*	Poaceae	Poales
SolyBFT1	Solyc01g009560.1.1	Phytozome	*Solanum lycopersicum*	Solanaceae	Solanales
SolyBFT2	Solyc01g009580.1.1	Phytozome	*Solanum lycopersicum*	Solanaceae	Solanales
SolyBFT3	Solyc03g026050.2.1	Phytozome	*Solanum lycopersicum*	Solanaceae	Solanales

SolyCEN1	Solyc06g074350.2.1	Phytozome	*Solanum lycopersicum*	Solanaceae	Solanales
SolyCEN2	U84140	NCBI	*Lycopersicon esculentum*	Solanaceae	Solanales
SolyCEN3	Solyc09g009560.1.1	Phytozome	*Solanum lycopersicum*	Solanaceae	Solanales
SolyFT1_1	Solyc05g055660.1.1	Phytozome	*Solanum lycopersicum*	Solanaceae	Solanales
SolyFT1_2	Solyc05g053850.2.1	Phytozome	*Solanum lycopersicum*	Solanaceae	Solanales
SolyFT1_3	Solyc11g008660.1.1	Phytozome	*Solanum lycopersicum*	Solanaceae	Solanales
SolyFT1_4	Solyc11g008640.1.1	Phytozome	*Solanum lycopersicum*	Solanaceae	Solanales
SolyFT1_5	Solyc11g008650.1.1	Phytozome	*Solanum lycopersicum*	Solanaceae	Solanales
SolyFT1_6	Solyc03g063100.1.1	Phytozome	*Solanum lycopersicum*	Solanaceae	Solanales
SolyMFT1	Solyc03g119100.1.1	Phytozome	*Solanum lycopersicum*	Solanaceae	Solanales
SolyMFT2	Solyc02g079290.2	Phytozome	*Solanum lycopersicum*	Solanaceae	Solanales
SotuBFT1	PGSC0003DMT400030575	Phytozome	*Solanum tuberosum*	Solanaceae	Solanales
SotuBFT2	PGSC0003DMT400030582	Phytozome	*Solanum tuberosum*	Solanaceae	Solanales
SotuBFT3	PGSC0003DMT400037143	Phytozome	*Solanum tuberosum*	Solanaceae	Solanales
SotuCEN	DQ307621	NCBI	*Solanum tuberosum*	Solanaceae	Solanales
SotuFT1_1	PGSC0003DMT400041725	Phytozome	*Solanum tuberosum*	Solanaceae	Solanales

SotuFT1_2	PGSC0003DMT400041726	Phytozome	*Solanum tuberosum*	Solanaceae	Solanales
StpuFT2C	TRINITY_DN58232_c0_g1_i1	UdeA	*Stelis pusilla*	Orchidaceae	Asparagales
StpuFT1B	TRINITY_DN29778_c0_g1_i1	UdeA	*Stelis pusilla*	Orchidaceae	Asparagales
StpuFT1C1	TRINITY_DN47332_c0_g1_i1	UdeA	*Stelis pusilla*	Orchidaceae	Asparagales
StpuFT1C2	TRINITY_DN64228_c0_g1_i1	UdeA	*Stelis pusilla*	Orchidaceae	Asparagales
StpuFT2A1	TRINITY_DN30350_c0_g1_i1	UdeA	*Stelis pusilla*	Orchidaceae	Asparagales
StpuFT2A2	TRINITY_DN30350_c0_g1_i2	UdeA	*Stelis pusilla*	Orchidaceae	Asparagales
StpuFT2A3	TRINITY_DN30350_c0_g1_i3	UdeA	*Stelis pusilla*	Orchidaceae	Asparagales
StreFT1_1	TRINITY_DN39864_c0_g1_i1	UdeA	*Streptosolen jamesonii*	Solanaceae	Solanales
StreFT1_2	TRINITY_DN25611_c0_g1_i1	UdeA	*Streptosolen jamesonii*	Solanaceae	Solanales
StreFT1_3	TRINITY_DN19473_c0_g1_i1	UdeA	*Streptosolen jamesonii*	Solanaceae	Solanales
StreMFT	TRINITY_DN72085_c0_g1_i1	UdeA	*Streptosolen jamesonii*	Solanaceae	Solanales
StSP	PGSC0003DMT400018307	Phytozome	*Solanum tuberosum*	Solanaceae	Solanales
StSP6A	PGSC0003DMT400060057	Phytozome	*Solanum tuberosum*	Solanaceae	Solanales
StSP9D	PGSC0003DMT400090526	Phytozome	*Solanum tuberosum*	Solanaceae	Solanales
TgFT1	MG121853.1	NCBI	*Tulipa gesneriana*	Liliaceae	Liliales
TgFT2	MG121854.1	NCBI	*Tulipa gesneriana*	Liliaceae	Liliales
TgFT3	MG121855.1	NCBI	*Tulipa gesneriana*	Liliaceae	Liliales

ToluFT1A	TRINITY_DN35395_c0_g1_i1	UdeA	*Tolumnia* Cherry red *x* Ralph yagh	Orchidaceae	Asparagales
ToluFT1C	TRINITY_DN13131_c0_g1_i1	UdeA	*Tolumnia* Cherry red *x* Ralph yagh	Orchidaceae	Asparagales
ToluFT2A1	TRINITY_DN13162_c0_g1_i1	UdeA	*Tolumnia* Cherry red *x* Ralph yagh	Orchidaceae	Asparagales
ToluFT2A2	TRINITY_DN13162_c0_g1_i2	UdeA	*Tolumnia* Cherry red *x* Ralph yagh	Orchidaceae	Asparagales
TraeFT2B	BT009051	NCBI	*Triticum aestivum*	Poaceae	Poales
TraeMFT	BT008995	NCBI	*Triticum aestivum*	Poaceae	Poales
TraeTFL1	AJ577366	NCBI	*Triticum aestivum*	Poaceae	Poales
VaplFT1A1	VPTC024845	ORCHIDSTRA	*Vanilla planifolia*	Orchidaceae	Asparagales
VaplFT1A2	VPTC003814	ORCHIDSTRA	*Vanilla planifolia*	Orchidaceae	Asparagales
VaplFT1A3	VPTC003875	ORCHIDSTRA	*Vanilla planifolia*	Orchidaceae	Asparagales
VaplFT1A4	VPTC023242	ORCHIDSTRA	*Vanilla planifolia*	Orchidaceae	Asparagales
VaplFT1A5	VPTC023243	ORCHIDSTRA	*Vanilla planifolia*	Orchidaceae	Asparagales
VaplFT1A6	VPTC021349	ORCHIDSTRA	*Vanilla planifolia*	Orchidaceae	Asparagales
VaplFT1B1	VPTC028579	ORCHIDSTRA	*Vanilla planifolia*	Orchidaceae	Asparagales
VaplFT1B2	VPTC002523	ORCHIDSTRA	*Vanilla planifolia*	Orchidaceae	Asparagales

VaplFT1B3	VPTC020362	ORCHIDSTRA	*Vanilla planifolia*	Orchidaceae	Asparagales
VaplFT1C1	VPTC001827	ORCHIDSTRA	*Vanilla planifolia*	Orchidaceae	Asparagales
VaplFT1C2	VPTC014381	ORCHIDSTRA	*Vanilla planifolia*	Orchidaceae	Asparagales
VaplFT1C3	VPTC023887	ORCHIDSTRA	*Vanilla planifolia*	Orchidaceae	Asparagales
VaplFT2A1	VPTC013244	ORCHIDSTRA	*Vanilla planifolia*	Orchidaceae	Asparagales
VaplFT2A2	VPTC021241	ORCHIDSTRA	*Vanilla planifolia*	Orchidaceae	Asparagales
VaplFT2A3	VPTC013243	ORCHIDSTRA	*Vanilla planifolia*	Orchidaceae	Asparagales
VaplMFT1	VPTC028836	ORCHIDSTRA	*Vanilla planifolia*	Orchidaceae	Asparagales
VaplMFT2	VPTC028837	ORCHIDSTRA	*Vanilla planifolia*	Orchidaceae	Asparagales
VaplTFL1_1	VPTC023176	ORCHIDSTRA	*Vanilla planifolia*	Orchidaceae	Asparagales
VaplTFL1_2	VPTC023149	ORCHIDSTRA	*Vanilla planifolia*	Orchidaceae	Asparagales
VashFT1B	Unigene8580_Va_fb	Orchidbase	*Vanilla shenzhenica*	Orchidaceae	Asparagales
VashTFL1	Unigene126478_Va_fb	Orchidbase	*Vanilla shenzhenica*	Orchidaceae	Asparagales
ViviBFT	DQ871593	NCBI	*Vitis vinifera*	Vitaceae	Vitales
ViviCEN	NM_001281000.1	NCBI	*Vitis vinifera*	Vitaceae	Vitales
ViviFT1	DQ504308.1	NCBI	*Vitis vinifera*	Vitaceae	Vitales
ViviMFT1	GSVIVT01008404001	Phytozome	*Vitis vinifera*	Vitaceae	Vitales
ViviMFT2	DQ871594	NCBI	*Vitis vinifera*	Vitaceae	Vitales

ViviMFT3	GSVIVT01003663001	Phytozome	*Vitis vinifera*	Vitaceae	Vitales
ViviTFL1_1	GSVIVT01033829001	Phytozome	*Vitis vinifera*	Vitaceae	Vitales
ViviTFL1_2	DQ871592	NCBI	*Vitis vinifera*	Vitaceae	Vitales
YubrFT1B	YBML_scaffold_2022499	1KP	*Yucca brevifolia*	Asparagaceae	Asparagales
YubrFT2A	YBML_scaffold_2030879	1KP	*Yucca brevifolia*	Asparagaceae	Asparagales
YubrFT2C	YBML_scaffold_2121017	1KP	*Yucca brevifolia*	Asparagaceae	Asparagales
ZemaFT1A1	Zm00008a021623	Phytozome	*Zea mays*	Poaceae	Poales
ZemaFT1A2	Zm00008a013824	Phytozome	*Zea mays*	Poaceae	Poales
ZemaFT1B1	GRMZM2G062052	Phytozome	*Zea mays*	Poaceae	Poales
ZemaFT1B2	GRMZM2G075215	Phytozome	*Zea mays*	Poaceae	Poales
ZemaFT1C	GRMZM2G158809	Phytozome	*Zea mays*	Poaceae	Poales
ZemaFT2B1	Zm00008a024302	Phytozome	*Zea mays*	Poaceae	Poales
ZemaFT2B2	Zm00008a030326	Phytozome	*Zea mays*	Poaceae	Poales
ZemaMFT1	BT016592	NCBI	*Zea mays*	Poaceae	Poales
ZemaTFL1	Zm00008a014804	Phytozome	*Zea mays*	Poaceae	Poales
ZemaZCN1	EU241917	NCBI	*Zea mays*	Poaceae	Poales
ZemaZCN10	EU241926	NCBI	*Zea mays*	Poaceae	Poales
ZemaZCN11	GRMZM2G117057	Phytozome	*Zea mays*	Poaceae	Poales
ZemaZCN12	EU241928	NCBI	*Zea mays*	Poaceae	Poales

ZemaZCN14	EU241929	NCBI	*Zea mays*	Poaceae	Poales
ZemaZCN15	EU241930	NCBI	*Zea mays*	Poaceae	Poales
ZemaZCN16	EU241931	NCBI	*Zea mays*	Poaceae	Poales
ZemaZCN17	EU241932	NCBI	*Zea mays*	Poaceae	Poales
ZemaZCN18	EU241933	NCBI	*Zea mays*	Poaceae	Poales
ZemaZCN19	EU241934	NCBI	*Zea mays*	Poaceae	Poales
ZemaZCN2	GRMZM2G156079	Phytozome	*Zea mays*	Poaceae	Poales
ZemaZCN20	EU241935	NCBI	*Zea mays*	Poaceae	Poales
ZemaZCN3	EU241919	NCBI	*Zea mays*	Poaceae	Poales
ZemaZCN5	EU241921	NCBI	*Zea mays*	Poaceae	Poales
ZemaZCN6	EU241922	NCBI	*Zea mays*	Poaceae	Poales
ZemaZCN8	EU241924	NCBI	*Zea mays*	Poaceae	Poales
ZemaZCN9	EU241925	NCBI	*Zea mays*	Poaceae	Poales
ZomaFT1_1	Zosma118g00560.1	Phytozome	*Zostera marina*	Zosteraceae	Alismatales
ZomaFT1_2	Zosma189g00010.1	Phytozome	*Zostera marina*	Zosteraceae	Alismatales
ZomaFT1_3	Zosma354g00060.1	Phytozome	*Zostera marina*	Zosteraceae	Alismatales
ZomaFT1_4	Zosma21g00670.1	Phytozome	*Zostera marina*	Zosteraceae	Alismatales
ZomaMFT	Zosma246g00290.1	Phytozome	*Zostera marina*	Zosteraceae	Alismatales

ZomaTFL1_1	Zosma165g00160.1	Phytozome	*Zostera marina*	Zosteraceae	Alismatales
ZomaTFL1_2	Zosma2g00820.1	Phytozome	*Zostera marina*	Zosteraceae	Alismatales
